# scHolography: a computational method for single-cell spatial neighborhood reconstruction and analysis

**DOI:** 10.1186/s13059-024-03299-3

**Published:** 2024-06-24

**Authors:** Yuheng C. Fu, Arpan Das, Dongmei Wang, Rosemary Braun, Rui Yi

**Affiliations:** 1 Driskill Graduate Program in Life Sciences, Northwestern University Feinberg School of Medicine, Chicago, IL 60611 USA; 2 Department of Pathology, Northwestern University Feinberg School of Medicine, Chicago, IL 60611 USA; 3 Robert H. Lurie Comprehensive Cancer Center, Northwestern University Feinberg School of Medicine, Chicago, IL 60611 USA; 4 Department of Molecular Biosciences, Northwestern University, Evanston, IL 60208 USA; 5 Department of Engineering Sciences and Applied Mathematics, Northwestern University, Evanston, IL 60208 USA; 6 Department of Physics and Astronomy, Northwestern University, Evanston, IL 60208 USA; 7 NSF-Simons Center for Quantitative Biology, Northwestern University, Evanston, IL 60208 USA; 8 Department of Dermatology, Northwestern University Feinberg School of Medicine, Chicago, IL 60611 USA

**Keywords:** Single-cell spatial transcriptomics, Spatial neighborhood analysis, Neural network, Deep learning, Stable matching neighbor

## Abstract

Spatial transcriptomics has transformed our ability to study tissue complexity. However, it remains challenging to accurately dissect tissue organization at single-cell resolution. Here we introduce scHolography, a machine learning-based method designed to reconstruct single-cell spatial neighborhoods and facilitate 3D tissue visualization using spatial and single-cell RNA sequencing data. scHolography employs a high-dimensional transcriptome-to-space projection that infers spatial relationships among cells, defining spatial neighborhoods and enhancing analyses of cell–cell communication. When applied to both human and mouse datasets, scHolography enables quantitative assessments of spatial cell neighborhoods, cell–cell interactions, and tumor-immune microenvironment. Together, scHolography offers a robust computational framework for elucidating 3D tissue organization and analyzing spatial dynamics at the cellular level.

**Supplementary Information:**

The online version contains supplementary material available at 10.1186/s13059-024-03299-3.

## Background

The cell is the basic building block of life. Tissues are composed of many heterogeneous cells, usually numbering in the millions to billions. Each cell occupies a unique location and plays specific roles that contribute to the physiological functions of the tissue. These functions include adhesion, proliferation, differentiation, and communication with other cells. The expression of genes within a cell not only determines its identity but also dictates its spatial location within a tissue. This relationship between gene expression and cell localization as well as tissue architecture has been supported by genetic studies, in which manipulation of gene expression can cause reproducible structural changes in tissues during development and homeostasis. However, it is challenging to map individual cells to 3-dimensional (3D) space and quantitively investigate the neighborhood of individual cells, based on their gene expression patterns [1–3]. The development of single-cell RNA sequencing (scRNA-seq) has permitted more accurate measurement of the transcriptome at the single-cell level [4]. More recently, spatial transcriptomic (ST) technologies have been developed to measure transcriptome and spatial information at the same time. There are generally two types of ST technologies. Imaging-based ST methods enable sub-cellular capturing resolution, built upon either in situ sequencing (ISS) [5, 6] or in situ hybridization (ISH) methods [7, 8]. Despite their high spatial resolution, imaging-based ST methods usually profile 100–1000 genes for each sample, well below the complexity of cellular transcriptome. On the other hand, sequencing-based ST methods, utilizing spatially barcoded transcript capture, allow whole-transcriptome expression profiling [9–11]. However, the resolution of sequencing-based ST is limited by the size of the micropatterned pixels, which can range from 10 to 100 μm and capture up to 20 cells. While methods with larger-size pixels have a lower resolution due to a mixing capture of transcriptomes from multiple cells within one pixel, methods with smaller-size pixels suffer from noise caused by lateral RNA diffusion [12] as well as technical dropout of transcripts. Furthermore, it is impossible to precisely match pre-printed pixels to individual cells within a slide. Collectively, the true single-cell resolution ST has yet to be established [1–3]. Computational methods, including cell-type deconvolution of spatial pixels, such as SPOTlight [13], spatialDWLS [14], STdeconvolve [15], and RCTD [16], and single-cell spatial charting methods, such as Seurat [17], Tangram [18], cytoSPACE [19], and Celltrek [20], have been developed to enhance the resolution of ST and gain new insights into tissue organization. However, these methods acquire the spatial information of ST pixels as 2D registration, which is dependent upon the sectioning angle of the reference slide. Single cells, which are profiled separately and sometimes not from the same donor of the ST data, are usually mapped back to the 2D spatial positions constrained by the reference slide, and this often leads to overpopulated spatial locations occupied by cells with the similar transcriptome. This approach limits the ability to identify cell neighbors and study cell–cell interactions at single-cell resolution. It remains unclear whether a 2D tissue slice contains the information to infer 3D tissue organization and whether it is possible to effectively learn such information from spatial transcriptomic datasets for functional investigation.

In this study, we aim to match single cells with their spatial neighbors, reconstruct tissue neighborhoods, and create 3D visualization of the reconstructed tissue to power the study of spatial dynamics of the transcriptome and tissue microenvironment. To address the limitations of current ST and computational methods, we have developed a new computational framework, scHolography. Our approach is based on three concepts. First, we reason that a distributed description of a spatial location, defined by the distance between one pixel to all other pixels within an ST reference, can more accurately capture the spatial identity of individual pixels than 2D coordinates alone. Furthermore, this inter-pixel spatial information can better capture the intrinsic organizing principles of the tissue. Second, we use neural networks to learn the transcriptome-to-space (T2S) transformation and implement the Gale-Shapley algorithm to identify Stable-Matching Neighbors (SMNs) of each query cell for reconstructing single-cell spatial neighborhoods and visualizing the reconstruction in 3D space. This approach also minimizes the possibility of assigning multiple similar cells to the same spatial location. Finally, we generate a quantitative description of cell cluster-specific microenvironment by computing the accumulative transcriptome of the nearest neighbors of spatially defined cell populations.

We benchmark scHolography against established single-cell spatial charting methods to demonstrate its accuracy in recapitulating biological tissue structure. We then validate our method with both sequencing-based ST platforms and imaging-based ST platforms. We further illustrate scHolography’s ability to predict 3D cell organization by extracting spatial information from 2D references. By applying scHolography to in-house generated human skin data and a recently published human skin cancer dataset [21] as well as a mouse kidney dataset [22], we show the utility of scHolography to reconstruct single-cell spatial neighborhoods, perform quantitative analyses of spatially defined cell clusters, and enhance the accuracy of cell–cell communication predictions. Together, scHolography not only provides a novel approach to enhance learning and spatial assignments of single-cell transcriptomic data without limiting the spatial charting to a fixed 2D reference tissue slice but also quantifies cellular microenvironment by integrating both neighbor cell type information and their associated transcriptome.

## Results

### scHolography learns inter-pixel spatial affinity and reconstructs single-cell tissue spatial neighborhoods

The scHolography workflow aims to resolve the spatial dynamics of tissue at single-cell resolution. One major goal of scHolography is to establish the transcriptome-to-space (T2S) projection, which helps to map single cells together with their spatial neighbors. While it is widely appreciated that scRNA-seq accurately measures the transcriptome and defines cellular states [23], it remains unclear which parameters could be used to define the spatial identities of a cell. Furthermore, current cell charting methods generally assign single cells back to the 2D ST reference section based on their 2D coordinates [19, 20]. These approaches assume that single cells are derived from a 2D tissue section, and this could lead to the loss of information for 3D tissue organization. We reason that the spatial positioning of cells within a 3D tissue structure is not solely determined by their 2D coordinates but is more accurately defined by cell–cell interactions within a microenvironment. Therefore, the spatial identity of a query SC data can be more accurately inferred through the study of cell–cell or pixel-pixel affinity represented in reference ST data instead of relying solely on the 2D coordinates of the reference.

**Fig. 1:**
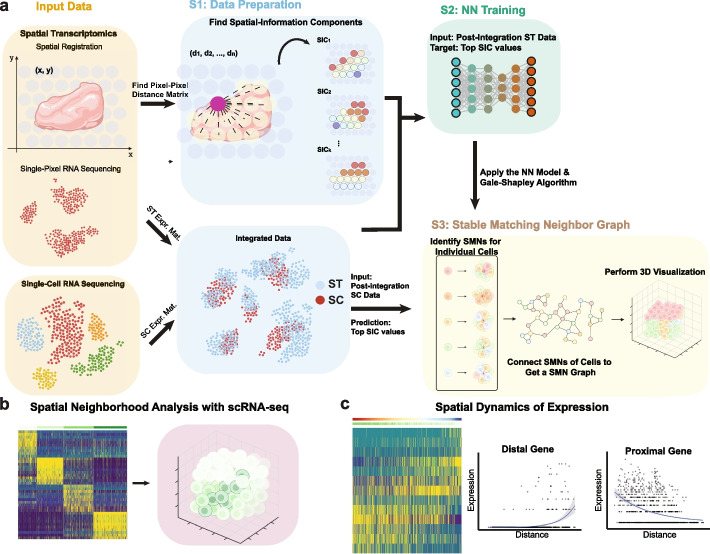
Overview of the scHolography workflow. **a** Three steps of the scHolography workflow. (1) scHolography takes in ST and SC expression data and ST 2D spatial registration data. Spatial-information components (SICs) are defined for the spatial registration data. ST and SC expression data are integrated. (2) Neural networks are trained with post-integration ST data as input and top SIC values as the target. (3) The trained neural networks are applied to post-integration SC data to predict top SIC values for SC. SIC values are referenced to infer cell–cell affinity and construct the stable matching neighbor (SMN) graph. The graph is visualized in 3D. **b** scHolography allows spatial neighborhood analysis. Cells are clustered according to their neighbor cell expression profile. **c** Based on inferred spatial distances among cells on the SMN graph, scHolography determines spatial dynamics of gene expression. The spatial gradient is defined as gene expression changes along the SMN distances from one cell population of interest to another

scHolography uses ST and SC data, obtained from tissue-type matched samples, as input (Fig. 1a Input Data). Specifically, scHolography acquires readily available 2D spatial registration from the reference ST data and generates a high-dimensional distance matrix of pairwise pixel-pixel distances from ST spatial registration. Principal component analysis (PCA) is then performed on the distance matrix to select top-ranked PCs and their corresponding values for downstream inferences. We name these top-ranked PCs of the distance matrix as spatial-information components (SICs) (Fig. 1a and Additional file 1: Fig. S1). Interestingly, these SICs capture distinct spatial patterns that are not only observable in the ST reference but also retained in the scHolography reconstruction visualizations (Additional file 1: Fig. S1a-f). To prepare data for model training, ST and SC expression data are then integrated into a shared manifold and SIC values for each ST pixel are defined (Fig. 1a Step1: Data Preparation; see the “Methods” section). Seurat CCA integration is chosen as the default method based on the result from a comparison in our simulated data (Additional file 1: Fig. S2), but different integration methods, such as Harmony, LIGER, and fastMNN, can also be used.

Next, scHolography trains neural networks to perform the T2S projection. scHolography utilizes post-integration ST expression data as training input and SIC values as training targets for generating the T2S projection model (Fig. 1a Step2: NN training). The trained model is then applied to SC data to infer spatial cell–cell affinity. The inferred spatial cell–cell affinity matrix is defined as the mean of cell–cell distance between predicted SIC values of runs. The closer distance is correlated with the higher spatial affinity. Finally, the Gale-Shapley algorithm is implemented to find Stable-Matching Neighbors (SMNs) for each cell by using the cell–cell affinity matrix as the matching utility. Cells are matched preferentially with those exhibiting higher spatial affinities through the application of the Gale-Shapley algorithm, chosen for its ability to yield stable matching pairs. This approach ensures that no cell pair would opt for an alternative match over the one currently assigned. The algorithm operates efficiently, employing a sequence of proposals and responses based on ranked preferences, leading to its polynomial time complexity O(n^2^), where *n* represents the total number of cells involved. Thus, scHolography uses the collective cell–cell affinity rather than a standalone coordinate to determine the spatial position of a single cell and ensure that every cell is assigned to a unique position, which is constrained by its SMNs. scHolography then visualizes 3D tissue organization by defining the cell–cell spatial connection with an undirected SMN graph and visualizing the SMN graph in 3D by the forced-directed Fruchterman-Reingold layout algorithm (Fig. 1a Step3: Stable Matching Neighbor Assignment).

In the reconstructed tissue, each cell is characterized by its unique spatial neighborhood, defined through the shortest path connecting individual cells on the SMN graph. Tissue spatial heterogeneity can be quantitatively studied not only through the examination of cell types within neighborhoods but also by clustering based on the collective expression profile of cells’ SMNs (see Fig. 1b). Furthermore, both local and global insights into tissue organization can be quantified by ordering cells according to their graph distances from a reference cell type and visualizing gene expression dynamics across the tissue’s spatial continuum (see Fig. 1c). Collectively, scHolography offers a comprehensive solution for 3D visualization of single-cell tissue structures, facilitating the identification of dynamic gene expression patterns and determining spatial cell heterogeneity.

### Benchmarking and validation of scHolography

**Fig. 2:**
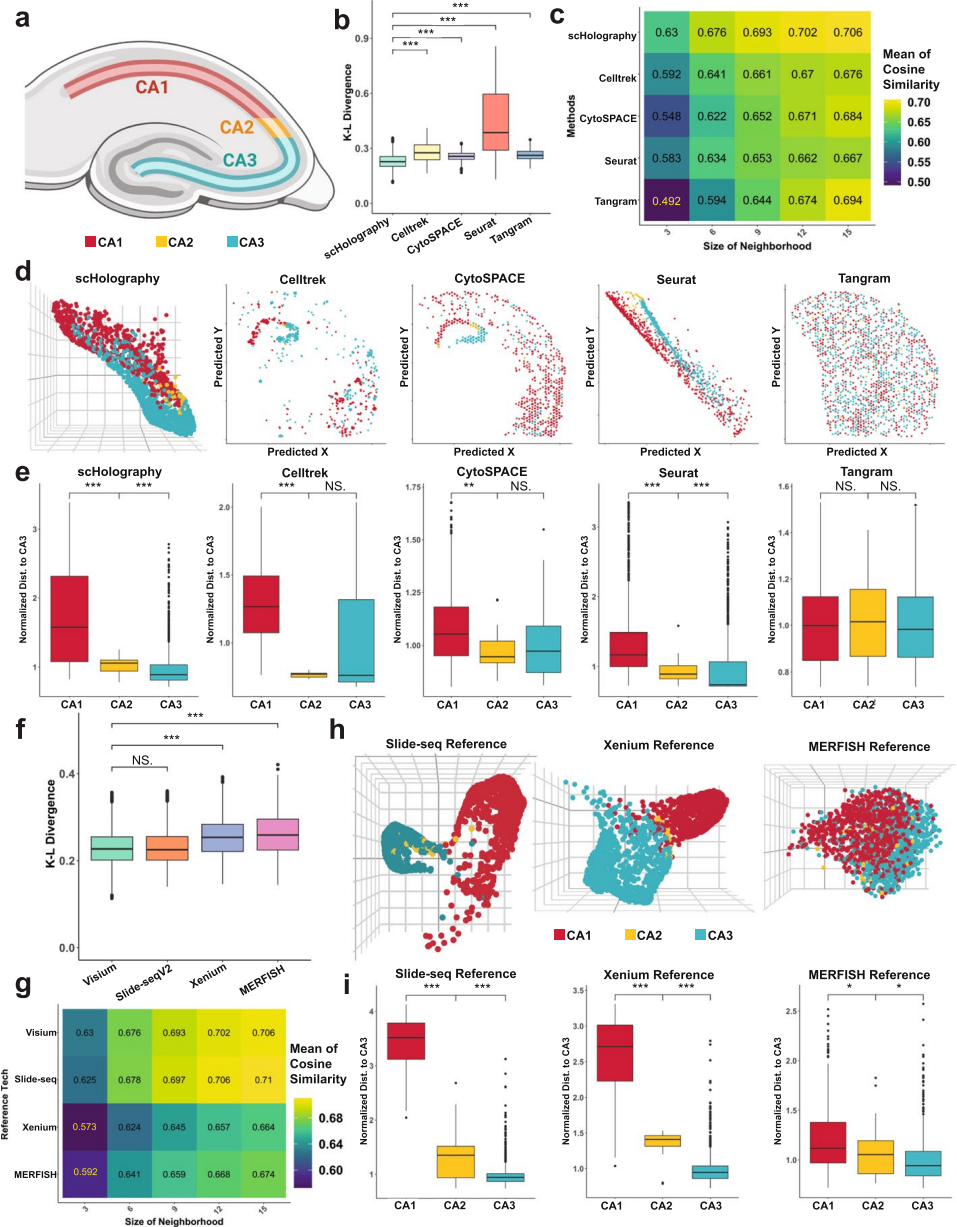
Benchmarking with other spatial cell charting methods. **a** Illustration of hippocampus CA subfields. **b** KL-divergence of spatial cell charting method predictions for simulated mouse hippocampus data as ground truth. **c** Heatmap for the mean of cosine similarity between method-predicted spatial neighborhood accumulated expression and simulated mouse hippocampus spatial neighborhood accumulated expression. The size of the neighborhood varies from 3 to 15 cells. **d** Visualization of scHolography, Celltrek, CytoSPACE, Seurat, and Tangram single-cell spatial charting results of a mouse hippocampus data. **e** Comparison of scHolography, Celltrek, CytoSPACE, Seurat, and Tangram results for predicted CA1, CA2, and CA3 cell distance to CA3 cells. Cell distances were normalized for each method by the mean distance between CA3 cells and CA3 cells. **f** KL-divergence of scHolography predictions for simulated mouse hippocampus data using different ST datasets as references. **g** Heatmap for the mean of cosine similarity between scHolography-predicted spatial neighborhood accumulated expression and simulated mouse hippocampus spatial neighborhood accumulated expression using different ST references. The size of the neighborhood varies from 3 to 15 cells. **h** Visualization of scHolography results of a mouse hippocampus data using Slide-seqV2, Xenium, and Merfish ST references. **i** Comparison of scHolography for predicted CA1, CA2, and CA3 cell distance to CA3 cells using Slide-seqV2, Xenium, and Merfish ST references. Cell distances were normalized for each method by the mean distance between CA3 cells and CA3 cells

The workflow of scHolography relies on the assumptions that the 2D ST reference dataset contains generalizable information for 3D tissue organization and that cell–cell affinity-based tissue reconstruction provides new insights into tissue organization. To validate scHolography, we first focused on the mouse hippocampus that contains spatially separated regions (Fig. 2a) and used two datasets: a simulated single-cell whole-transcriptome dataset of the mouse hippocampus region with spatial registration information obtained from the Vizgen platform (Vizgen MERFISH Mouse Brain Receptor Map; see the “Methods” section) and scRNA-seq data of the mouse hippocampus [24]. We applied scHolography to both datasets to reconstruct their spatial neighborhoods and visualize the inferred structure in 3D from a 10X Visium mouse brain ST reference data (10X Visium Mouse Brain Coronal Sect. 1 FFPE data). It is worth mentioning that the reference ST slice covers a larger brain region rather than restricted to the hippocampus region whereas the scRNA-seq data were obtained from hippocampus cell populations. We compared the results of scHolography with those from spatial cell charting methods, including Celltrek, CytoSPACE, Seurat, and Tangram. The benchmarking of the methods is based on comparisons using two key metrics: (1) K-L divergence, measuring the discrepancy between the predicted spatial distribution patterns of cells and their ground-truth counterparts, and (2) the average cosine similarity, assessing the alignment between the accumulated expression profiles within the predicted spatial neighborhoods and those within the ground-truth neighborhoods. While K-L Divergence measures a global spatial reconstruction quality, the average cosine similarity measures the accuracy of local neighborhood recapitulation (see the “Methods” section). Across both metrics, scHolography outperforms other methods, achieving the lowest K-L divergence and the highest average cosine similarity (Fig. 2b, c, n.s. (not significant): *p* > 0.05; *p ≤ 0.05; **p ≤ 0.01; ***p ≤ 0.001; ****p ≤ 0.0001, the same convention applies to all figures). Furthermore, in a focused analysis of the intricate Cornu Ammonis (CA) regions within the hippocampus using annotated scRNA-seq data, only scHolography and Seruat accurately delineate the spatial orders among the CA subfields—CA1, CA2, and CA3 (Fig. 2d–e). This precision contrasts with the difficulties faced by Celltrek, CytoSPACE, and Tangram. These methods encounter challenges due to the ST reference data covering a broader brain area than the scRNA-seq data. This discrepancy leads to non-specific spatial assignments and the erroneous classification of single cells, which should be confined to the hippocampus, across the entire reference space (Fig. 2d). These findings illustrate a critical limitation of 2D spatial cell charting techniques, particularly evident in instances of mismatched regions between ST and SC datasets. To examine the influence of mismatched regions on different computational methods, we performed additional benchmarking using a sub-region of the 10X Visium data that more closely aligns with the simulated SC region. While all evaluated methods showed improved performance on this matched subset, scHolography still demonstrated its superior ability to accurately predict global spatial distribution and effectively differentiate between cell types (Additional file 1: Fig. S3). Despite the dependence of scHolography’s predictions on random seed settings, the consistency of results across various seeds (Additional file 1: Fig. S4a) underscores the robustness of scHolography. Moreover, scHolography enhances model reliability by providing training and validation loss curves of each run and implementing early stopping mechanisms to minimize the risk of overfitting (Additional file 1: Fig. S4b).

Because the core function of scHolography is to generate a T2S projection based on ST and SC transcriptomic measurement, it should be readily applicable for any ST platforms that generate high-dimensional transcriptomic data. To verify its applicability, we next applied scHolography to ST data from diverse platforms, including sequencing-based Slide-seqV2, and imaging-based 10X Xenium and MERFISH. Overall, scHolography successfully delineated tissue structures and accurately assigned single cells to their appropriate spatial neighborhoods, effectively distinguishing between the CA1, CA2, and CA3 hippocampal subfields (Fig. 2f–i). Notably, sequencing-based ST datasets from 10X Visium and Slide-seqV2 yielded better results in terms of K-L Divergence and average cosine similarity (Fig. 2f, g). This enhanced performance can likely be attributed to the deeper transcriptome profiling provided by sequencing-based methods than their imaging-based counterparts.

### scHolography generates 3D spatial representation from 2D references

**Fig. 3:**
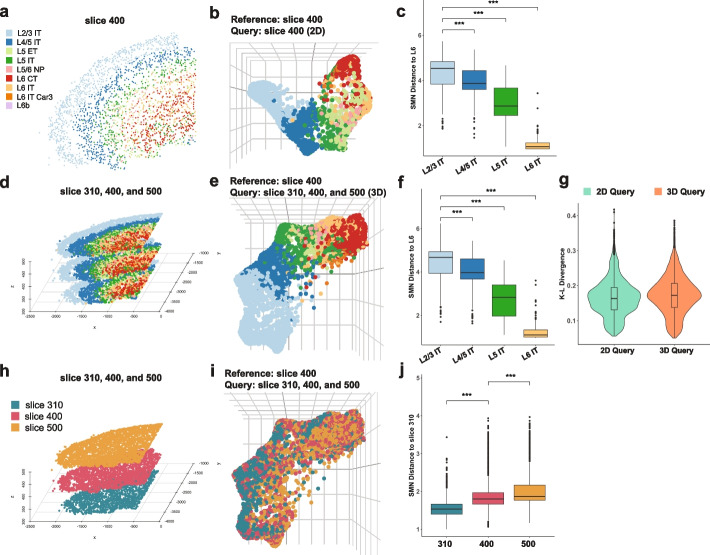
scHolography effectively reconstructs 3D tissues from 2D reference. **a** 2D plot of Merfish mouse cortex data (slice 400). **b** 3D visualization of Merfish slice 400 scHolography reconstruction result (prediction reference: slice 400; 2D query: slice 400). **c** SMN distances from L2/3 IT, L4/5 IT, L5 IT, L6 IT to L6 IT in slice 400 scHolography prediction. **d** Stacked-2D plot of Merfish mouse cortex data (slice 310, 400, and 500). **e** 3D visualization of Merfish slice 310, 400, and 500 combined scHolography reconstruction result (prediction reference: slice 400; 3D query: combined slice 310, 400, and 500). **f** SMN distances from L2/3 IT, L4/5 IT, L5 IT, L6 IT to L6 IT in slice 310, 400, and 500 combined scHolography prediction. **g** Comparison of KL-divergence for scHolography 2D and 3D query results both using 2D reference. **h** 3D plot of Merfish mouse cortex data (slice 310, 400, and 500) colored by the slice. **i** 3D visualization of Merfish slice 310, 400, and 500 combined scHolography reconstruction result (prediction reference: slice 400; 3D query: combined slice 310, 400, and 500) colored by the slice. **j** SMN distances from slice 310, 400, and 500 to slice 310 in slice 310, 400, and 500 combined scHolography prediction

To confirm the ability of scHolography to infer 3D tissue architecture from 2D ST references, we used recently published mouse cortex datasets generated by MERFISH, an imaging-based technique, from serial sections [25]. We first selected a single 2D ST slice (slice 400) from the MERFISH dataset for both reference and query in scHolography reconstruction (Fig. 3a, b). To quantitatively evaluate scHolography’s performance, we calculated the SMN distances from various cortical pyramidal neuron layers to the layer 6 (L6) neurons. The results agreed with expected biological patterns: L2/3 neurons were furthest from L6, with L4/5 and L5 neurons progressively closer, and L6 neurons the closest (Fig. 3c). This result confirms scHolography’s ability to recapitulate the stereotypical structure of cortical tissue in 2D.

We next applied scHolography to a composite dataset created by stacking slices 310, 400, and 500 from the MERFISH series, with slice 400 serving again as the reference (Fig. 3d). Considering these slices as serial sections from the same sample allowed us to treat the combined dataset as 3D data. The resulting visualizations (Fig. 3e) and layer distance quantifications (Fig. 3f) confirm scHolography’s ability to reconstruct 3D tissue architecture from 2D references, retaining accurate biological structure. Furthermore, comparing the K-L divergence from 2 and 3D queries revealed scHolography’s consistent performance (Fig. 3g). Interestingly, the analysis of distances among cells within each layer underscored scHolography’s precision in capturing subtle spatial differences. The SMN distances for cells in the composite dataset to slice 310 displayed an ascending trend corresponding to the order of slices 310, 400, and 500 (Fig. 3h–j).

To further validate the robustness of scHolography in generating 3D spatial representation from 2D data, we applied scHolography to two MERFISH samples, with Sample 1 comprising six slices and Sample 2 comprising five slices. We used the PASTE algorithm [26] to vertically integrate the slices within each sample, establishing this stacked-2D compilation as the benchmark for ground truth. For each sample, we conducted scHolography reconstruction twice, using either the bottommost or the uppermost slice as the reference point, respectively. Importantly, the fidelity of both reconstructions was consistently high, regardless of the chosen reference slice (Additional file 1: Fig. S5-6). Taken together, these results demonstrate that scHolography can extract cell–cell spatial affinity information from 2D ST references and effectively visualize 3D tissue organization.

### scHolography recapitulates global and local spatial organization of human skin

**Fig.4:**
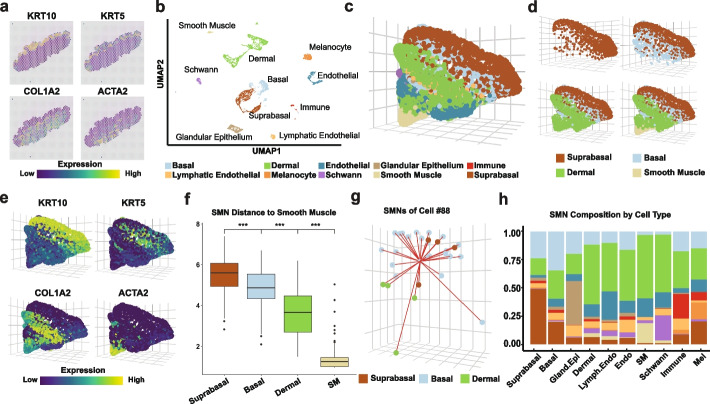
scHolography recapitulates the spatial organization of human skin. **a** Spatial feature plots of markers for four major cell types of human foreskin ST data (*KRT10*, suprabasal cell; *KRT5*, basal cell; *COL1A2*, dermal cell; *ACTA2*, smooth muscle cell). **b** UMAP plot of human foreskin scRNA-seq data. **c** 3D visualization of all cell types in scHolography human foreskin reconstruction. **d** 3D visualization of four major cell types in scHolography human foreskin reconstruction. **e** scHolography 3D feature plot of marker genes for 4 major cell. **f** SMN distances between 4 major foreskin cell types and smooth muscle cells (suprabasal cells *n* = 1118; basal cells *n* = 804; dermal cells *n* = 1529; smooth muscle cells *n* = 119). Boxplots show the median with interquartile ranges (IQRs) and whiskers extend to 1.5 × IQR from the box. Two-sided Wilcoxon tests are performed. **g** scHolography 3D plot of Cell #88 and its first-degree neighbors. **h** SMN cell type composition plot of each human foreskin cell type

To test the ability of scHolography to reconstruct tissue organization across different tissue types, we turned to freshly isolated human foreskin samples, whose 3D spatial organization and cell heterogeneity are well appreciated [27, 28]. We generated a 10X Visium ST dataset from a sagittal section of donor #1. This ST dataset captured 659 pixels with a median sequencing depth of 156,332 reads/pixel. By plotting with markers for major skin cell types, we confirmed that our ST data capture all major cell types in the skin, including epithelium, dermal, endothelial and smooth muscle cells (Fig. 4a). We also generated SC data, obtained from a different donor, donor #2, which captured 6425 cells with a mean sequencing depth of 136,235 reads/cell, and 5450 cells passed our filtering with the Seurat package [29]. Unsupervised clustering identified major populations of epithelial and dermal cell types (Fig. 4b). We also detected PECAM1 + endothelial cells, MGST1 + glandular epithelium, CD74 + immune cells, PROX1 + lymphatic endothelial cells, PMEL + melanocytes, MPZ + Schwann cells, and TAGLN + smooth muscle cells (Fig. 4b and Additional file 1: Fig. S7a-b).

We applied scHolography to the SC data to reconstruct the human foreskin at single-cell resolution by using the Visium ST data as the reference (Fig. 4c). scHolography reconstruction recapitulated stereotypical positions of major cell types, reflected by both cell type annotation and gene marker expression in the reconstructed 3D structure (Fig. 4d, e). For example, suprabasal epithelial cells, marked by KRT10^hi^ expression, were located at the outermost layer of the 3D structure, and KRT5^hi^ basal epithelial cells were located beneath the suprabasal cells and sandwiched between the suprabasal epithelial cells and dermal fibroblasts (Fig. 4d, e). The ACTA2^hi^ smooth muscle cells were located at the bottom of the reconstructed 3D tissue, consistent with the stereotypical cell organization of the skin (Fig. 4d, e).

The quantitative measurement of cell–cell distance, inferred by scHolography as the SMN distance, allows the study of tissue architecture based on spatial distance. It enabled us to analyze the distance between individual cell layers. We calculated the SMN distance between suprabasal, basal, dermal, and smooth muscle cells to smooth muscle cells. Not only were the differences highly significant between each cell type (Mann Whitney Wilcoxon test, *p* < 2.22e − 16), but also the spatial order agreed with stereotypical tissue organization such that suprabasal cells were furthest away from smooth muscle cells, followed by basal cells and fibroblasts (Fig. 2f). Furthermore, because scHolography reconstructed SMN graph designates up to 30 stable-matching neighbors to individual cells as their SMNs (Fig. 4g), we can determine the neighborhood composition for each cell type in the skin by accumulating the SMN cell type information for all cells from each cell type (Fig. 4h). For basal, suprabasal, and glandular epithelial cells, the most abundant neighbors to each cell type were themselves as expected. Notably, fibroblasts often emerged as the most abundant neighbors for cell types that were localized in the dermis, including endothelial cells, lymphatic endothelial cells, and Schwann cells (Fig. 4h). These observations are consistent with the high heterogeneity of dermal fibroblast cells and their complex interactions with other cell types [30, 31]. Together, scHolography reveals spatial cell heterogeneity based on their neighbor cell composition.

### Cell type composition analysis identifies transitioning cells during epidermal differentiation

**Fig. 5:**
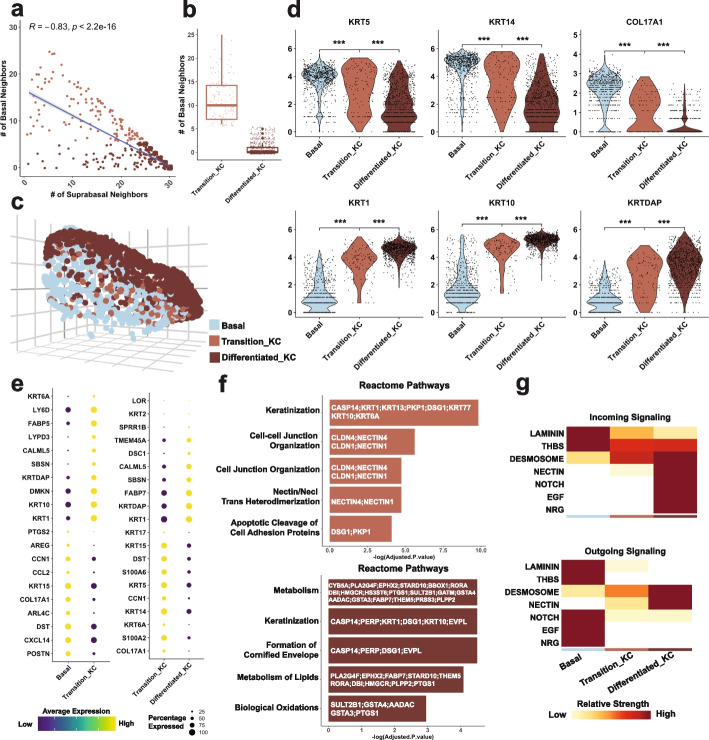
scHolography defined SMNs reflect cellular transition in skin epidermal differentiation. **a** The number of suprabasal SMNs and basal SMNs for each suprabasal cell. **b** The number of basal SMNs of each transition KC or differentiated KC. **c** 3D visualization of basal cells, transition KCs, and differentiated KCs in scHolography reconstruction. **d** Violin plots of epithelial progenitor and differentiation markers. Progenitor markers: KRT5, KRT14, COL17A1. Differentiation markers: KRT1, KRT10, KRTDAP. **e** Expression dot plots of top 10 upregulated and downregulated genes comparing basal cells vs. transition KC (left) or transition KC vs. differentiated KC. **f** Reactome pathway enrichment analysis on upregulated genes in transition KC compared to basal cells (top) and on upregulated genes in differentiated KC compared to basal cells (bottom). **g** Relative incoming (top) and outgoing (bottom) signaling strengths of CellChat inferred significant signaling for basal cell, transition KC, and differentiated KC clusters

Epidermal differentiation is a dynamic process coupling with spatial cues, where the innermost basal cells, consisting of undifferentiated progenitors, attach to the basement membrane (BM), and differentiating cells delaminate from the BM, remodel their adhesion with neighboring basal cells, and move upward to outer layers as they embark on terminal differentiation to form the protective barrier of the skin [32]. To determine whether scHolography reconstruction recapitulates spatial cell organizations, we investigated the spatial cell neighborhood of epithelial cells defined by scHolography. By plotting the number of basal cell neighbors against the number of suprabasal neighbors for all suprabasal cells, we observed a strong negative correlation among suprabasal cells (*R* =  − 0.83, *p* < 2.22e − 16) (Fig. 5a), which indicates that cell composition of the neighborhood could demarcate cellular states of these differentiating cells. Indeed, we readily separated suprabasal cells into two populations: (1) a transitioning keratinocyte population (transition KC) that is defined as suprabasal cells having more basal cell neighbors (more than 1.5 × IQR above the third quartile of the number of basal neighbors of all suprabasal cells) and (2) a terminally differentiated keratinocyte population (differentiated KC) that is defined as suprabasal cells having more suprabasal neighbors (Fig. 5b). 3D visualization of suprabasal and basal cells demonstrated a spatial mixing between transition KC and basal cell populations, whereas the terminally differentiated KC forms a more uniform, outermost layer of the skin (Fig. 5c). Gene expression analysis provided a quantitative view of the transitioning process from basal cells to transition KC to differentiated KC with stepwise decreased progenitor markers (Additional file 2: Table S1-4), including *KRT5*, *KRT14*, *and COL17A1*, and increased differentiation markers, including *KRT1*, *KRT10*, *and KRTDAP* (Fig. 5d). Notably, the downregulation of BM associated genes, such as COL17A1 and COL7A1, is more precipitous than intermediate filament, such as KRT5 and KRT14. Pairwise differentially expressed gene analysis demonstrated that although the transition KC population has higher differentiation marker expression of *KRT1 and KRTDAP* than basal cells, the differentiated KC population has much higher *KRT1*, *KRT10*, *and KRTDAP* than transition KC, along with additional well-studied terminal differentiation markers *LOR*, *KRT2*, and *DSC1* [33] (Fig. 5e). In addition, Reactome pathway enrichment analysis [34] identified *Keratinization* pathway commonly enriched for both transition KC and differentiated KC, whereas *Metabolism*, *Formation of Cornified Envelope*, *Metabolism of Lipids*, and *Biological Oxidations* pathways were uniquely enriched in terminally differentiated KC (Fig. 5f), consistent with drastically increased lipid and cornified envelope production in these barrier layers. Finally, we examined the expression patterns of the components of Notch signaling, which is known to promote epidermal differentiation [35, 36], in reconstructed basal and transition KC cell layers. Indeed, Notch ligands, including *JAG2* and *DLL1*, are highly enriched in basal cells and strongly downregulated in transition KC (Additional file 2: Table S1), whereas *NOTCH3* receptor and canonical Notch targets, such as *HES2* and *HES4*, are strongly upregulated in transition KC (Additional file 2: Table S2). The high granularity of gene expression patterns across these epithelial layers highlights the fidelity of scHolography-based reconstruction.

The higher spatial resolution of basal and suprabasal cells as well as their neighbors allows us to discern distinct cell–cell communication patterns among three epidermal populations by using CellChat analysis [37]. By incorporating spatial neighborhood information of the epidermal cells, we pinpointed that BM-associated signaling events, such as those mediated by Laminin and THBS, are largely confined within the basal cells. Interestingly, differentiation-associated signaling events, such as those mediated by desmosomes [38, 39], are gradually increased from the basal cells to transition KC and peaking in terminally differentiated cells (Fig. 5g). Furthermore, Notch signaling, which is known to promote epidermal differentiation [35, 36], shows binary patterns between basal and terminally differentiated cells (Fig. 5g). These results not only validate scHolography reconstruction of epidermal layers but also demonstrate the utility of scHolography for studying spatial gene expression patterns.

### scHolography reveals spatial heterogeneity in the dermis of human skin

**Fig. 6:**
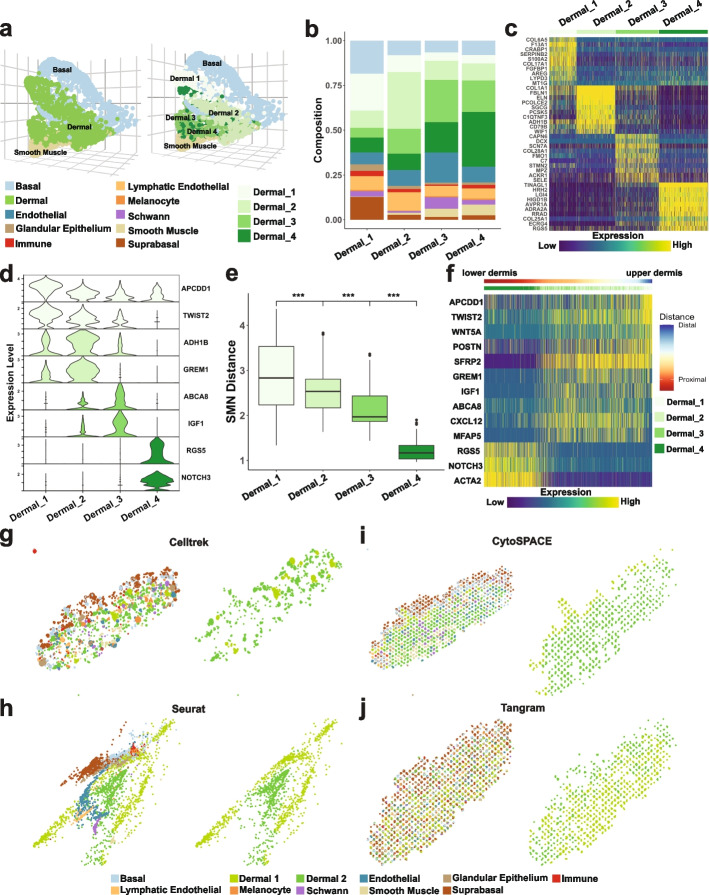
scHolography defined spatial neighborhoods reflect the heterogeneity of human skin fibroblast. **a** Spatial neighborhood analysis for human skin dermal cells. Four distinct neighborhoods Dermal 1–4 are identified based on the clustering of the accumulated expression profile of dermal SMNs. **b** Cell type composition of Dermal 1–4. **c** Heatmap of top 10 differentially expressed genes for Dermal 1–4 accumulated SMN expression profile. **d** Violin plots of Dermal 4 differentially expressed genes in scRNA-seq data for all dermal spatial neighborhoods. **e** SMN distances between Dermal 1–4 and Dermal 4 cells. **f** Expression heatmap of spatially dynamic genes of human dermal cells proximal (left) and distal (right) to smooth muscle cells. Dermal cells are ordered, from left to right, in increasing SMN distance to smooth muscle cells. **g**–**j** Single-cell spatial charting results of Celltrek, CytoSPACE, Seurat, and Tangram (left of each panel) and dermal cell spatial neighborhoods identified based on Celltrek, CytoSPACE, Seurat, and Tangram results (right of each panel)

With the ability of scHolography to reconstruct tissue organization with single cells, it raises the possibility of investigating spatial cell heterogeneity with spatially integrated transcriptomes. To do so, we accumulated the expression profile of cell neighborhood for each dermal cell and identify dermal cell subtypes by using distinct transcriptome expression and spatial distribution with the *findSpatialNeighborhood* function (see the “Methods” section). Four distinct dermal spatial neighborhoods (Dermal1–4) were identified (Fig. 6a–c). Notably, these spatially defined dermal cell populations not only show complex but distinct cell-type composition (Fig. 6b) but also have distinct transcriptome (Fig. 6c). Importantly, scHolography identified spatial neighborhoods differed from the Seurat clusters of scRNA-seq alone (Additional file 1: Fig. S3c). Further scRNA-seq expression analysis on dermal cells from different spatial neighborhoods identified Dermal 1 as papillary dermal cells with high *APCDD1* and *TWIST2* expression, Dermal 2 as reticular dermal cells with high *ADH1B* and *GREM1* expression, Dermal 3 as endothelial and pericyte-interacting dermal cells with high *ABCA8* and *IGF1* expression, and Dermal 4 as pericytes with high *RGS5* and *NOTCH3* expression (Fig. 6d), consistent with experimentally identified derma cell populations [30]. Quantification of the virtual SMN distance (Fig. 6e) and marker gene expression patterns (Fig. 6f) across the reconstructed dermis showed a decreasing trend in the distance between Dermal 1–4 and Dermal 4 cells and unique patterns for each marker, respectively, in line with the notion that papillary dermal cells are located at the upper dermis whereas reticular dermal cells are located at the lower dermis [27, 31]. As a comparison, we also conducted the spatial neighborhood analysis on Celltrek, Seurat, CytoSPACE, and Tangram results, using the same query SC data and reference ST data. While these methods have different spatial charting results, spatial neighborhood analysis from all four methods only identified two spatial neighborhoods (Fig. 6g–j). Combined with the accumulated transcriptome of cell neighborhood obtained from each method, Celltrek and Seurat distinguished pericyte from other dermal cells, while CytoSPACE and Tangram only identified lower and upper dermal cells (Additional file 1: Fig. S7d-g). We also explored the ability of SPOTlight [13], a spot-deconvolution-based method, for spatial neighborhood analysis, combing with the *BuildNicheAssay* approach described by Seurat V5 [40] on the same SC query data and ST reference data. However, this spot-deconvolution approach failed to capture the differences in the dermal regions, specifically the pericyte, papillary, and reticular regions (Additional file 1: Fig. S8). Taken together, these results demonstrate the superior capability of scHolography not only in reconstructing 3D tissue structures but also in delineating spatial cellular heterogeneity.

### scHolography recapitulates mouse kidney spatial organization

**Fig. 7:**
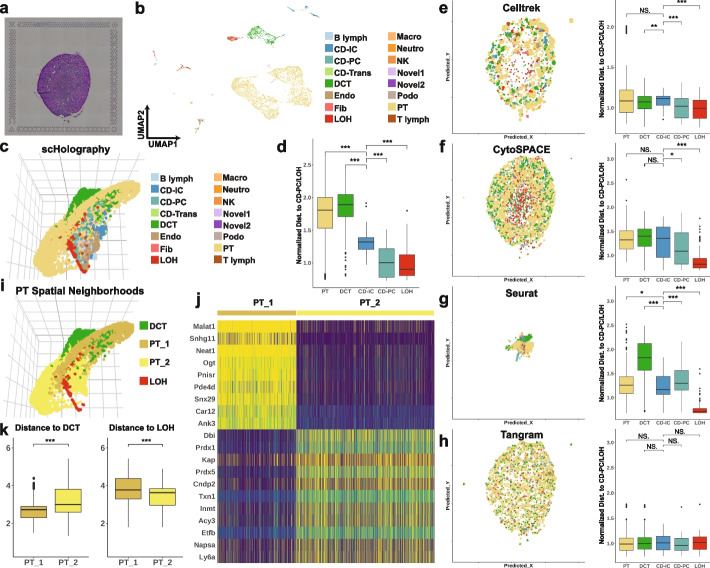
scHolography recapitulates mouse kidney spatial organization. **a** H&E image of mouse kidney coronal section. **b** UMAP plot of mouse kidney scRNA-seq data. **c** 3D visualization of mouse kidney scHolography reconstruction result. **d** Boxplots for scHolography predicted PT, DCT, CD-IC, CD-PC, and LOH cell distance to CD-PC and LOH cells. Cell distances were normalized for each method by the mean distance from CD-PC and LOH cells to the two cell types. Two-sided Wilcoxon tests are performed. **e**–**h** Comparison of Celltrek, CytoSPACE, Seurat, and Tangram mouse kidney prediction results. Left panel: Visualization of prediction outcome. Right panel: Predicted PT, DCT, CD-IC, CD-PC, and LOH cell distance to CD-PC and LOH cells. Cell distances were normalized for each method by the mean distance from CD-PC and LOH cells to the two cell types. Two-sided Wilcoxon tests are performed. **i** Spatial neighborhood analysis for mouse kidney PT cells. Two distinct neighborhoods are identified based on the clustering of the accumulated expression profile of PT SMNs. **j** Heatmap of top marker genes for PT_1 and PT_2 accumulated SMN expression profile. **k** Violin plot of SMN distance between two PT spatial neighborhood cells and DCT cells (left). Violin plot of SMN distance between two PT spatial neighborhood cells and LOH cells (right). Two-sided Wilcoxon tests are performed

To further examine the performance of scHolography for spatial analysis of tissue organization, we next turned to a complex tissue, the kidney. The kidney plays a vital role in filtering waste from the blood and excreting them through urine, featuring a multifaceted structure segmented into distinct zones that each contributes uniquely to urine production. These spatially and functionally distinct zones include the outer layer cortex, the outer medulla, and the inner medulla, moving from the exterior to the interior of the kidney [41]. Using a combination of micro-dissection and scRNA-seq approaches, it has been shown that proximal tubule (PT) and distal convoluted tubule (DCT) cells are predominantly found within the cortex, whereas collecting duct principal cells (CD-PC) and loop of Henle (LOH) cells are primarily found in the inner medulla. Collecting duct intercalated cells (CD-IC) are distributed throughout all three layers [41]. Applying scHolography to a mouse kidney dataset [22] with a 10X Visium reference (Fig. 7a, b), we generated a detailed reconstruction of the spatial arrangements of PT, DCT, CD-IC, CD-PC, and LOH cells within the kidney structure (Fig. 7c, d), which reflect the anatomical organization of these zones. In comparison, the results from other spatial mapping methods only partially recapitulated the distinct organization of the kidney (Fig. 7e–h). Leveraging the SMN neighborhood and single-cell transcriptome, we next performed spatial neighborhood analysis of PT cells and identified two distinct spatial neighborhoods (Fig. 7i, j). Specifically, the first neighborhood, PT_1, is situated closer to DCT cells, while the second, PT_2, lies closer to LOH cells, highlighting the intricate spatial relationships within the kidney’s cellular architecture (Fig. 7k). Notably, each neighborhood has a group of highly enriched genes, which can provide markers for validation and functional analysis.

### scHolography dissects tumor-immune microenvironment in squamous cell carcinoma

**Fig. 8:**
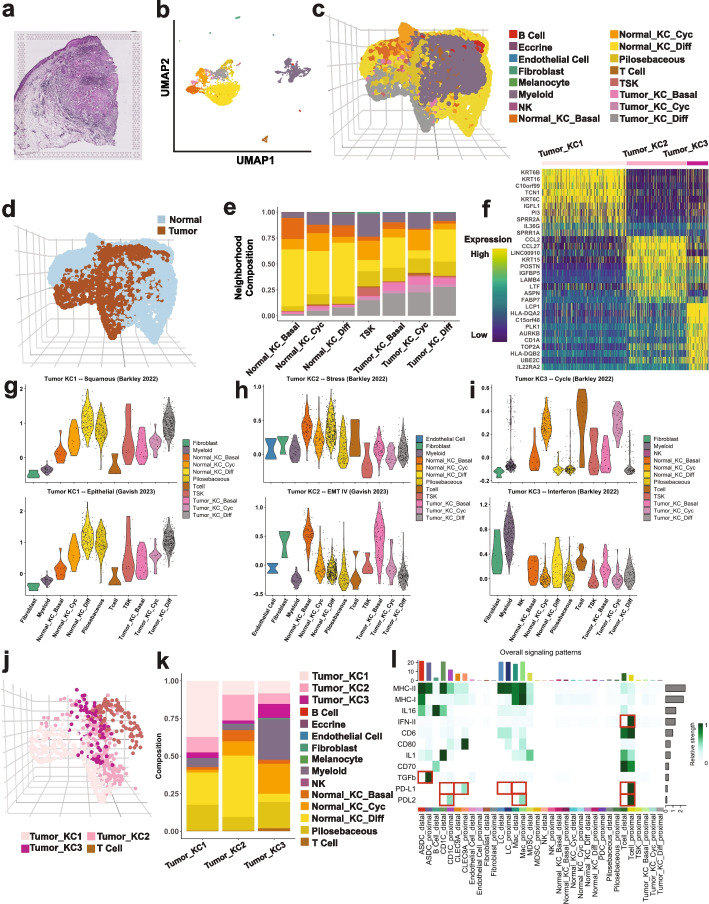
scHolography dissects the spatial tumor-immune microenvironment in squamous cell carcinoma. **a** H&E image of Patient 6 rep 1 cSCC ST sample. **b** UMAP plot of Patient 6 rep 1 scRNA-seq data. **c** 3D visualization of human cSCC scHolography reconstruction result. **d** 3D visualization of cSCC scHolography result colored by disease conditions**. e** Cell type composition plot for SMN of keratinocyte cell types in cSCC. **f** Heatmap of top 10 differentially expressed genes for accumulated SMN expression profile of each scHolography identified tumor keratinocyte spatial neighborhood. Three spatial neighborhoods, Tumor KC1-3, are identified. **g** Violin plots of the squamous score (*n* = 44 genes, Barkley et al. [44]) in SMNs of Tumor KC1 cells (top), and violin plots of the epithelial score (*n* = 29 genes, Gavish et al. [43]) in SMNs of Tumor KC1 cells (bottom). **h** Violin plots of the stress score (*n* = 88 genes, Barkley et al. [44]) in SMNs of Tumor KC2 cells (top), and violin plots of the EMT score (*n* = 32 genes, Gavish et al. [43]) in SMNs of Tumor KC2 cells (bottom). **i** Violin plots of the cell cycle score (*n* = 271 genes, Barkley et al. [44]) in SMNs of Tumor KC3 cells (top), and violin plots of the interferon score (*n* = 99 genes, Barkley et al. [44]) in SMNs of Tumor KC3 cells (bottom). **j** 3D visualization of Tumor KC 1–3 and T cells in cSCC scHolography result. **k** Cell type composition plot for SMN of Tumor KC 1–3 in cSCC. **l** Overall signaling strengths inferred by CellChat for different cell types in cSCC. Based on scHolography, each cell type is split into (1) distal and (2) proximal groups. Cells in the distal group are not SMNs of any tumor keratinocyte cells, while cells in the proximal group are SMNs of at least one tumor keratinocyte cell

To test spatial neighborhood analysis in a more complex cellular environment, we applied scHolography to previously published patient- and site-matched normal and diseased human cutaneous squamous cell carcinoma (cSCC) ST and SC datasets [21] (Fig. 8a–d). To compare the spatial organization of normal skin and cSCC, we focused on the neighborhood composition of each keratinocyte (KC) cell type. As expected, different KC cell types, including both normal and tumor KCs, had a wide range of variation in the neighborhood composition (Fig. 8e). Consistent with previous results, while basal, cycling, and differentiated KCs were identified in both normal and tumor regions, tumor KCs also contained a unique cluster, named tumor-specific keratinocytes (TSKs) [21]. TSKs are enriched at the leading edge of the tumor, and these cells show invasive and immunosuppressive features [21]. Interestingly, the cell neighborhoods of normal KCs, including basal, cycling, and differentiated KCs, were largely composed of themselves or other normal KCs (Fig. 8e). In sharp contrast, the neighborhood profiles of tumor KCs were more complex with notably increased shares of immune cells, including T cells and myeloid cells (Fig. 8e). Pilosebaceous cells were also enriched as the neighboring cells for all tumor KCs, consistent with the notion that hair follicle stem cells serve as the cell of origin for tumor KCs [42]. Interestingly, TSKs showed the most diverse composition of cell neighborhoods with the highest share of T cells and myeloid cells, consistent with their location at the leading edge of the tumor and robust interactions with immune cells. Furthermore, when we quantified the virtual distance between different tumor KCs and T and myeloid cells, TSKs, along with tumor cycling KCs, were predicted to be closest to T cells and myeloid cells whereas differentiated tumor KCs were furthest away from these immune cells (Additional file 1: Fig. S9a). Together, scHolography reconstruction recapitulated spatial features described in these SCC samples.

To unravel the complexity of tumor KC and immune cell interaction, we conducted a spatial neighborhood analysis on all tumor KCs by integrating the transcriptome profile. Three tumor spatial neighborhoods, Tumor KC1/2/3, were identified by differentially expressed genes (DEGs) among each spatially defined cell cluster (Fig. 8f). Enrichment analysis for DEGs [43–45] of distinct tumor KC neighborhood revealed significantly enriched signatures for squamous and epithelial senescence genes in Tumor KC1, stress and epithelial-to-mesenchymal transition (EMT) genes in Tumor KC2, and cell cycle and interferon genes in Tumor KC3 (Additional file 1: Fig. S9b-d). Similar to dermal cells in normal skin, transcriptome-based tumor KC classification was different from spatial neighborhood-based tumor KC classification. Among these three tumor spatial neighborhoods, differentiated tumor KCs were predominantly presented in Tumor KC1, basal tumor KCs were highly enriched in Tumor KC2, and TSKs and cycling tumor KCs were highly abundant in Tumor KC3 (Additional file 1: Fig. S10a). Because each spatial neighborhood was reconstructed with single-cell transcriptome, we further analyzed transcriptomic features within each spatial neighborhood. Notably, differentiated normal KC and tumor KC contributed significantly to the squamous and epithelial signatures of Tumor KC1 neighborhood whereas TSK showed high heterogeneity (Fig. 8g). In Tumor KC2 neighborhood, normal KCs, including basal, cycling, and differentiated cells, contributed significantly to the stress signature whereas tumor basal cells contributed most significantly to the EMT signature (Fig. 8h). As expected, both normal and tumor cycling KCs contributed significantly to the cell cycle signature of Tumor KC3. Interestingly, however, T cells showed strong heterogeneity in their cell cycle status within Tumor KC3 neighborhood (Fig. 8i), reflecting both exhausted and proliferative T cell populations. Furthermore, myeloid cells and, to a lesser extent, fibroblasts contributed significantly to the interferon signature (Fig. 8i). These analyses provide a quantitative view of different tumor KC neighborhoods.

We next examined the spatial distribution of different cell types. Tumor KC3 was predicted to be proximal to T cells and myeloid cells, compared with Tumor KC1 and KC2 (Fig. 8j and Additional file 1: Fig. S10b-c). Consistent with these molecular features, the neighborhood cell-type composition revealed a highly dynamic immune cell landscape with a high share of T cell and myeloid cells within the Tumor KC3 neighborhood (Fig. 8k). Because of the high myeloid cell composition within Tumor KC3 neighborhood, we further dissected the myeloid cells into several subsets, including AXL + SIGLEC6 + dendritic cells (ASDCs), CD1C + dendritic cells (CD1Cs), CLEC9A + dendritic cells (CLEC9As), Langerhans cells (LCs), macrophages (Macs), myeloid-derived suppressor cells (MDSCs), and plasmacytoid dendritic cells (PDCs), and compared their differential spatial affinity with tumor KCs and T cells (Additional file 1: Fig. S10d). Interestingly, PDCs, followed by ASDCs and CLEC9As, showed a closer SMN distance to both T cells and tumor KCs (Additional file 1: Fig. S10d-e). In addition to their role in generating significant levels of IFNα during viral infections, PDCs have been demonstrated to have a regulatory impact on various types of cancer and contribute to tumor progression in SCCs [46, 47]. Notably, PDCs contribute to immunosuppression via the activation of regulatory T cells and through impaired IFNα production [48]. ASDCs, or pre-DCs, are precursors of conventional DCs (cDCs) that can differentiate into (1) CLEC9A + cDC1 [49] and (2) CD1C + FCER1 + DC, or cDC2 [50]. Of note, cDC1 plays a role in antitumor immunity by tumor-specific CTLs induction and T cell recruitment [51]. Collectively, the identification of both anti-tumor and pro-tumor DCs proximal to Tumor KCs and T cells reveals the complex landscape of the tumor immune microenvironment.

Having reconstructed single-cell spatial neighborhoods with their associated transcriptome, we next examined cellular crosstalk within and outside the tumor microenvironment. We divided each non-tumor KC cell type into two groups: (1) the proximal group, containing cells belonging to the cell neighborhoods of any tumor KCs; (2) the distal group, containing cells outside the tumor KC neighborhoods. We performed CellChat analysis by incorporating the inferred spatial information (Fig. 8l and Additional file 1: Fig. S10f). Strikingly, immune cell types, including ASCD, CD1C, CLEC9A, LC, Mac, MDSC, and T cells, demonstrated polarized signaling directions and strengths when integrating their spatial localization information into the analysis. For example, while we observed high anti-tumor type II Interferon (IFN-II) signaling in T cells that are proximal to tumor KCs, Programmed cell death ligand 1 (PD-L1) and Programmed cell death ligand 2 (PD-L2) signaling are also high in tumor KC-proximal T cells compared to those distal T cells, which agreed with the immunosuppressive microenvironment around the tumor defined by the original publication [21]. Besides T cells, scHolography also detected an increased level of PD-L1 and PD-L2 in other immune cells, including CD1C, CLEC9A, and LC, providing more putative targets of immunosuppressive signaling. Moreover, protumor transforming growth factor-beta (TGFb) signaling is identified as high in tumor KC-proximal ASDCs compared to distal ASDCs. This result suggests that ASDCs may interact with FOXP3-expressing regulatory T cells to maintain immune tolerance, in response to TGFb. These results demonstrate the utility of scHolography for studying complex cell microenvironments and examining cell–cell interactions in a spatially defined manner.

## Discussion

In this study, we have established a new computational solution to spatial transcriptomics, which defines the spatial identity of single cells with a focus on cell neighborhood, generates a neural network-based T2S projection for spatial neighborhood reconstruction, and determines spatial cell heterogeneity. The limitation of using 2D coordinates to describe spatial identity is that the location of each pixel is simply determined by its coordinates. Thus, the interconnectedness of cell organization patterns within a tissue is not captured. Consequently, the use of 2D coordinates fails to capture the information for intricate spatial organization of cells or accurately delineate their proximal relationships within a tissue. Instead of employing 2D coordinates, scHolography uses an inter-pixel distance matrix to characterize the spatial identity of cells within a tissue. This approach leverages the information from all pixels in the tissue to define the spatial identity of individual pixels, thereby retaining critical information about tissue organization. Additionally, the high-dimensional nature of the inter-pixel distance matrix in scHolography facilitates the application of neural networks and deep learning algorithms, enabling precise mapping of a cell’s transcriptome to its spatial location. Interestingly, the T2S projection learned from low-resolution ST data is applicable to SC data without any cell-type deconvolution. We further demonstrated the superior performance of scHolography over a spot deconvolution-based method. We speculate the driving force for the better performance is that scHolography uses single-cell data as the query and spatial data as the reference, and each cell is assigned to a unique location. Thus, scHolography can make inferences on single cells with a higher resolution than spot deconvolution. In contrast, decomposition methods treat spatial data as the query and single-cell data from each cell cluster as the reference. This strategy loses transcriptome heterogeneity detected by high-dimensional single-cell data within each cell cluster. Leveraging stable matching neighbor assignment, scHolography successfully reconstructs 3D tissue organization of relatively simple tissues such as the human foreskin and complex tissues such as mouse kidney and human skin cancer. These findings establish a link between a cell’s transcriptome and its spatial localization within tissue—a relationship that can be learned using scHolography. The employment of SMNs and the Gale-Shapley algorithm for 3D spatial assignment effectively mitigates the clustering effect, wherein similar single cells disproportionately overpopulate specific spatial spots or neighborhoods.

While scHolography is an effective tool for spatial transcriptomics, several limitations could influence its performance. We note that tissue heterogeneity is reflected by not only diverse cell types but also different cell densities. In this regard, scHolography reconstructs the spatial neighbor graph using a predetermined number of neighbors’ fixed neighbor numbers, which could not adjust to different cell densities within a tissue. This effect is evident in the analysis of the simulated mouse hippocampus data, where scHolography shows better performance in regions of higher cell density compared to areas with lower density (Additional file 1: Fig. S11). For future development, integrating cell segmentation data and using information from multiple spatial slices could provide important insights into cell density. Future advances in the spatial resolution of ST methods and integrated learning from multiple ST datasets from the same tissue are also poised to refine the accuracy of deep learning-based reconstruction further. These approaches hold promise for finetuning the performance of scHolography across both imaging- and sequencing-based spatial transcriptomics.

## Conclusion

Taken together, we present scHolography, a novel computational approach to spatial transcriptomics, which effectively addresses the limitations of traditional spot deconvolution and spatial charting methods by utilizing an inter-pixel distance matrix to define the spatial affinity of cells. This method paves the way for more accurate studies of tissue architecture and cellular interactions. Furthermore, scHolography opens avenues for exploring the impact of genetic and epigenetic perturbations on the spatial configuration of cells within tissue. The genetic information encoded in the genome not only dictates cellular states but also fundamentally shapes tissue and organismal architecture. By using scHolography, this can provide invaluable insights into how alterations in gene expression influence the structural integrity of tissues and organisms. Collectively, these investigations hold the promise to uncover new paradigms in cell–cell communication and tissue organization across various biological processes, including development, homeostasis, wound healing, aging, and disease.

## Methods

### The scHolography workflow

#### Step 1: Data preparation

scHolography takes ST and SC expression data and ST 2D spatial registration data as input. scHolography first integrates the ST and SC expression data with the Seurat reference-based integration method [17]. From integrated data, scHolography obtains matrices ${X}_{p,q}$ and ${Y}_{c,q}$ where $X$ are the top expression principal components (default = 32) for SC data and $Y$ are the top expression principal components (default = 32) for ST data. Next, for 2D spatial registration data associated with ST data, scHolography calculates pairwise Euclidean distance matrix ${D}_{p,p}$ between spatial spots. Top *d* principal components (default = 32) are then found for the distance matrix *D*, and we rename the principal components as spatial-information components (SICs). The SIC matrix is denoted as ${D}_{p,d}{\prime}$.

#### Step 2: Neural network training

scHolography trains a neural network with ${X}_{p,q}$ as the predictor matrix and ${D}_{p,d}{\prime}$ as the predicting target. The neural network functions are powered by the Keras package [52] and have the following architecture:

**Table:** 

Name	Operation	Number of Features	Dropout	Batch Normalization	Activation	Input
input	-	32	X	X	-	-
FC-1	FC	32	0.2	✓	Leaky ReLU	input
FC-2	FC	32	0.2	✓	Leaky ReLU	FC-1
FC-3	FC	8	0.2	✓	Leaky ReLU	FC-2
FC-4	FC	32	0.2	✓	Leaky ReLU	FC-3
output	FC	32	X	X	ReLU	FC-4
Optimizer	Adam		# of Epochs	500	Loss	MSE
Learning Rate	0.001		α	0.00005		
Leaky ReLU slope	0.2		Patience	20		
Batch Size	32					

The network architecture is optimized with a bottleneck layer to compress information for fitting. The trained neural network will be applied to ${Y}_{c,q}$ to predict cell-specific spatial-information score ${P}_{c,d}$ corresponding to each previously identified SIC values. Based on the predicted score matrix $P$, scHolography calculates cell–cell distance and normalizes for individual cells to obtain an inferred cell–cell affinity matrix ${A}_{c,c}$. Step 2 will be repeated for $n$ times (default = 30) and the median of each ${A}_{c,c}$ entry will be found across repeated runs to reduce the prediction variance. Denote the resulting affinity matrix as ${\widehat{A}}_{c,c}$ and the variance of each ${A}_{c,c}$ entry across repetitions as the learning variance matrix $M$. Under the default setting, outlier cells with high total variance across repeated runs (more than 1.5 × IQR above the third quartile) will be filtered out.

#### Step 3: Stable matching neighbor assignment

From the affinity matrix ${\widehat{A}}_{c,c}$, scHolography applies the Gale–Shapley algorithm to find $k$ stable matching neighbors for every single cell via the MatchingR package [53]. The affinity matrix is used as the utility for matching. Each cell functions simultaneously as a host cell and as a potential guest neighbor cell to other host cells in the Gale-Shapley matching process. Following each of the *k* rounds of the Gale–Shapley assignment, a host cell is paired with a guest cell, forming a stable matching pair. To ensure optimal distribution of matches, the affinity between successfully matched cell pairs is reduced after each round. This reduction in affinity prioritizes previously unmatched cells in subsequent rounds of assignments, thereby optimizing the allocation of guest cells to different host cells throughout the matching process. Note that not all cells will be assigned $k$ (default = 30) stable neighbors. Fewer neighbors will be assigned if there is not enough stable matching. The final stable matching results are represented in an unweighted graph. We name the graph as a stable matching neighbor (SMN) graph. Once the SMN graph is determined, scHolography constructs the 3D visualization with the forced-directed Fruchterman-Reingold layout algorithm of the graph [54]. By default, the random seed is set to 60,611 for all steps above.

### findDistance function

If $a, b$ are single cells within an SMN graph, we define the SMN distance between them by

$$d\left(a,b\right)=\text{the length of the shortest path from }a\text{ to }b\text{ on the SMN graph}$$

The findDistance function then enables the distance measurement of individual cells to a given cell type or cluster of cells on the SMN graph. We define the distance between a cell $x$ and a cell group $A$ by

$$D\left(x,A\right)=\frac{\sum_{i=1}^{k}d({a}_{i},x)}{k},$$

${a}_{1},\dots ,{\text{a}}_{\text{k}}$ are the $k$ nearest cells (default = 30) from group $A$ to $x$ measured by SMN distance.

### findSpatialNeighborhood function

The findSpatialNeighborhood function aims to define distinct spatial neighborhoods and study single-cell spatial heterogeneity in a transcriptome-spatial integrated manner. First, the function decides the number of distinct neighborhoods to define from scHolography inferred query cell spatial distribution. The silhouette coefficient optimizes the number of spatial neighborhoods. The accumulated SMN expression profile of SMNs for each query cell is defined as the sum of the scRNA-seq count of all SMNs of the query cell. The accumulated SMN expression matrix is normalized using the Seurat SCTransform function, and the PCAs of the normalized matrix are input to define spatial neighborhoods using K-means clustering with the optimized cluster number. Differentially expressed genes are found for both accumulated SMN and single-cell expressions of each spatial neighborhood using the Seurat FindAllMarkers function.

### scHolographyNeighborCompPlot function

The scHolographyNeighborCompPlot function plots the SMN composition for each query cell type or annotation. The function also identifies enriched neighbor types for query cells with significance levels using the Wilcoxon test.

### Generation of simulated mouse *hippocampus* data

To evaluate the accuracy and robustness of scHolography, we simulated a single-cell ST dataset with known single-cell 2D spatial information using publicly available mouse hippocampus scRNA-seq data with annotations, referred to as true SC data [55], and the hippocampus region (3000 < *x* ≤ 7000, 1000 < *y* ≤ 4000, randomly subsampled to 8000 cells) of Vizgen mouse brain ST data (Vizgen MERFISH Mouse Brain Receptor Map, Slice 2 Replicate 1). Since the Vizgen ST dataset contains 483 genes, we use the scRNA-seq to simulate single-cell ST data from Vizgen ST by imputing variable genes from scRNA-seq data—the simulated mouse hippocampus SC data containing 8000 cells and 3000 genes. For the imputation, the Seurat FindTransferAnchors and TransferData functions were used with weight.reduction = “PCA” and dims = 1:50. The ST data used for benchmarking is 10X Visium Mouse Brain Coronal Sect. 1 FFPE data. Simulated SC, true SC data, and ST data were all normalized using Seurat SCTransform.

### Benchmarking analysis with simulated SC and SC data

We performed our benchmarking study against Celltrek, CytoSPACE, Seurat, and Tangram to evaluate the performance of scHolography. These methods were considered for benchmarking because their common goal of single-cell spatial charting aligns with scHolography major function. Yet, we still would like to point out the fundamental differences between scHolography and these methods: while other methods focus on assigning 2D coordinates to single cells, scHolography is innovative in finding spatial neighbors to single cells. Because of this innovation, it is hard to compare scHolography with spot-level decomposition methods like RCTD and cell2location. We did our benchmarking analysis using both simulated SC and SC mouse hippocampus data. With ground truth 2D coordinates, simulated SC data allows quantified evaluation under the two metrics described below. The true SC data with annotation allows targeted and quantified prediction evaluation for cell types of interest.

Two metrics, K-L divergence and cosine similarity, were used for methods evaluation.

1. K-L divergence. K-L divergence is a statistic describing the difference between two distributions. We first defined spatial distribution as:

$${P}_{ij}=\frac{{D}_{ij}}{\sum_{1}^{N}{D}_{ij}}$$

where ${D}_{ij}$ denotes the inferred spatial distance between cell *i* and cell *j*, and *N* is the total number of cells. Then, the K-L divergence of each cell is calculated as:

$$KL({a}_{i}|\left|{b}_{i}\right)=\sum_{j=0}^{N}{a}_{ij}\times log\frac{{a}_{ij}}{{b}_{ij}}$$

where ${a}_{i}$ is the predicted spatial distribution, and ${b}_{i}$ is the ground truth spatial distribution.

2. Cosine similarity. Cosine similarity is used to assess the similarity between the accumulated transcriptome of the predicted nearest spatial neighborhood of size *k* around cell *i* vs. the ground truth nearest spatial neighborhood of size k around cell *i*.

$$\text{Cosine similarity}_{i,k}= \frac{A \bullet B}{\left|\left|A\right|\right|\left|\left|B\right|\right|}$$

where A and B are the predicted and ground truth accumulated transcriptome matrix, respectively.

A scHolography
  Default settings of scHolography were used for both simulated SC and true SC prediction from ST. The 3D visualization coordinates of scHolography results were used for benchmarking and comparison with other methods.
B Celltrek
  We ran Celltrek with following parameters: intp_pnt= 2000, nPCs=30, ntree=1000, dist_thresh=999, top_spot=1, spot_n= 100, repel_r=20 with 20 iterations. This setting aimed to reduce the number of unmapped cells for a fair comparison.
C CytoSPACE
  Default settings of CytoSPACE were used with the lap_CSPR solver for both simulated SC and true SC prediction from ST. Since cells can be mapped to non-unique spots of ST spatial 2D coordinates, for cells with multiple 2D assignments, the mean of each 2D coordinate is calculated and used for the final predicted coordinates. Also, since cells can be assigned for the same 2D coordinates, a random small jitter was added to coordinate to distinguish the cells with the same assignment.
D Seurat
  Default settings of Seurat were used with normalization.method = “SCT” and dims = 1:30 with the FindTransferAnchors function followed by the TransferData function to transfer the 2D coordinates from ST spatial data.
E Tangram
  Default Tangram settings, as defined in the Tangram tutorial, were used for predicting spatial alignment from ST data to SC data, covering both simulated and actual SC datasets. Based on the probability matrix generated by Tangram, which estimates the likelihood of cells being located in various spots, we assigned each cell to the spot where it had the highest probability of being found. Again, since cells can be assigned for the same 2D coordinates, a random small jitter was added to coordinate to distinguish the cells with the same assignment.

To demonstrate that scHolography can take both sequencing- and imaging-based ST data as prediction reference, we extended our benchmarking using ST reference from Slide-seqV2, 10X Xenium, and Merfish, besides the previously discussed 10X Visium mouse brain ST data.

A Slide-seqV2
  We used published mouse hippocampus Slide-seqV2 data [56]. We randomly subsampled 8000 cells from the data as the scHolography prediction reference. Data was normalized by Seurat SCTransform.
B 10X Xenium
  We used 10X Genomics Fresh Frozen Mouse Brain for Xenium Explorer Demo data. We included only cells from the hippocampus region (2500< *x* ≤ 4700, 2000< *y* ≤ 3250, randomly subsampled to 8000 cells) as scHolography prediction reference. Data was normalized by Seurat SCTransform.
C Merfish
  We used published anterior preoptic mouse brain region Merfish data [57]. Specifically, we took a slice from Animal 1 with a Bregma 0.06 mm. We included only cells in the Merfish data with the Centroid Y coordinate of no less than 4000 as scHolography prediction reference. Data was normalized by Seurat SCTransform.

### Benchmarking analysis with different integration methods

Harmony [58], LIGER [59], and fastMNN [60] results, in addition to Seurat CCA integration, on simulated mouse hippocampus data were used to prepare SC and ST data prior to scHolography reconstruction. This analysis was conducted under SeuratWrappers V0.2.0, rliger V1.0.1, and harmony V1.2.0 with default settings, followed by default scHolography NN Training and Stable Matching Neighbor Graph steps using scHolography trainHolography function.

### Benchmarking analysis with region-matched simulated SC and ST data

Compared to unmatched analysis, region-matched benchmarking analysis used the simulated SC data and a subfield of 10X Visium Mouse Brain Coronal Sect. 1 FFPE data (25 < row < 55, 35 < col < 75). Both simulated SC data and 10X Visium reference data were centered around the mouse hippocampus region. scHolography, Celltrek, CytoSPACE, Seurat, and Tangram predictions and benchmarking analysis were conducted as the simulated SC analysis using the full 10X Visium Mouse Brain reference.

### Validation analysis with Merfish mouse cortex data

Three consecutive slices, slices 310, 400, and 500, of Animal 1 were acquired from the Merfish mouse cortex data [25]. The three 10-µm slices correspond to the positions at 310 µm, 400 µm, and 500 µm of the sample. For the stacked-2D sample analysis, the coordinates of slices 310 and 500 were normalized using slice 400 as the reference, such as the coordinate median in all three samples being the same as slice 400. Seurat SCTransform normalized data from all three slices.

### Merfish multiple-slice sample processing and analysis

We used previously published data from the anterior preoptic region of the mouse brain, obtained from the MERFISH platform. From this dataset, we selected Sample 1 and 2, both derived from Animal 1, specifically focusing on the central z-plane for our analysis. Cell centroids were determined by using the “terra” package. For each sample, we subsampled 1500 cells from each slice. To assemble a stacked 2D structure from Sample 1 and 2, we used the “PASTE” package [26] for pairwise slice alignment within each sample. The data underwent normalization through Seurat’s SCTransform command. For Sample 1, which comprises six slices, we performed two separate reconstructions: one using slice 10 and another using slice 500 as references. Similarly, for Sample 2, which contains five slices, reconstructions were carried out twice, using slice 620 and slice 1020 as references, respectively.

### Human foreskin sample collection and sequencing

Neonatal foreskins from Donors 1 and 2 were collected as discarded, deidentified tissue under IRB protocol #STU00009443 of the Northwestern University Skin Biology and Diseases Resource-based Center. Donor 1 sample was punched by an 8-mm punch and embedded in the sagittal direction into an FFPE block by SBDRC.

For the scRNA-seq experiment, fresh human foreskin specimens from Donor 2 were cut into 4 mm × 4 mm pieces. The dermal fat layer was trimmed off from the bottom. Then, the skin was floated on 2 mL of dispase in a 6-well plate and incubated at 37 °C for 1 h. The epidermis was separated from the dermis and trypsinized for 12 min at 37 °C to get the epidermal single-cell suspension. For the dermis part, it was further cut into smaller pieces, then incubated with 0.25% collagenase I in 2 mL HBSS for 1 h at 37 °C. Collagenase-treated pieces were trypsinized for 10 min at 37 °C. The tissue was then dissociated by pipetting and single-cell suspension was obtained. Epidermal and dermal cells were combined at a 1:1 ratio and used as scRNA-seq input materials. The Single-Cell Chromium 3′ v3 kit from 10 × Genomics was used for single-cell library preparation. Final scRNA-seq libraries were sequenced on an Illumina NovaSeq-6000 system.

The Cell Ranger v.6.0.0 was applied to align reads to the human reference GRCh38 (GENCODE v32/Ensembl 98), and a gene expression matrix was obtained. The Seurat package v4 was used for data processing and visualization, and the default settings were applied unless otherwise noted. Cells with fewer than 200 or more than 7000 unique feature counts were filtered. Besides, cells with more than 15% of mitochondrial counts were also filtered. The normalization was performed by sctransform [61]. Variable genes were found with the FindVariableFeatures function and PCA was conducted by RunPCA. The top 30 PCs were selected with ElbowPlot for downstream analyses. Cell clusters were identified by FindNeighbors and FindCluster functions at a resolution of 0.5. RunUMAP was used for 2D visualization. DE genes were identified by the FindAllMarkers function and the top DE genes for each cluster were considered for cell identity annotation.

For ST experiments, RNA quality was first checked for the sample. Total RNA was isolated from a 20-µm Donor 1 FFPE block section using Qiagen RNeasy FFPE Kit following the manufacturer’s instructions. RNA quality was evaluated using the DV200 assay on Agilent Bioanalyzer. The sample was used for library preparation after confirming the quality of RNA is desired based on DV200 (DV200 > 50%; DV200 = proportion of RNA fragments with > 200 nucleotides in length).

A 5-µm section was sliced from Donor 1 FFPE block, placed on 10X Genomics Visium Spatial Gene Expression Slide v1, deparaffinized, and H&E stained under the manufacturer’s protocol. The sample was placed in the A1 region. A 5-µm replicate was acquired and was placed in the B1 region. Brightfield images were acquired at 20 × magnification using a Nikon Ti2 widefield microscope system for 2 h. Images were processed with the Nikon NIS-elements software. The samples were then decrosslinked, and the human whole transcriptome probe panel was hybridized to the RNA from the decrosslinked tissue. Next, probes were ligated, released from the tissue, extended, and indexed. All these steps followed the manufacturer’s instructions. For library construction, 17 cycles of sample index PCR were performed.

Final ST libraries were sequenced on an Illumina NovaSeq-6000 system. The Space Ranger v.1.3.1 was applied to align reads to the human reference GRCh38 (GENCODE v32/Ensembl 98). The Seurat package v4 was again used for data processing and visualization, and the default settings were applied unless otherwise noted. The normalization was performed by sctransform [61]. Variable genes were found with the FindVariableFeatures function and PCA was conducted by RunPCA. The top 32 PCs were selected for downstream analyses. Pixel clusters were identified by FindNeighbors and FindCluster functions at a resolution of 0.5. RunUMAP was used for 2D visualization. DE genes were identified by the FindAllMarkers function.

### Human foreskin data analysis

Donor 2 scRNA-seq data were reconstructed by scHolography using Donor 1 ST data as the reference. Default scHolography settings were used. For human skin epithelial differentiation analysis, based on the number of basal SMNs, we separated suprabasal cells into two populations. Outlier suprabasal cells with a larger number of basal SMNs (more than 1.5 × IQR above the third quartile) were annotated as transition KC, and suprabasal cells were annotated as differentiated KC otherwise. Differentially expressed genes were found separately (1) for basal and transition KC and (2) for transition KC and differentiated KC. Genes enriched in comparison (1) transition KC and comparison (2) differentiated KC were used respectively for the Reactome analysis (*p*.adjusted < 0.05). The CellChat analysis [37] was performed to dissect ligand-receptor interactions for suprabasal and basal cells in Donor 2 scRNA-seq data with default settings on cells with basal, transition KC, and differentiated KC annotation. Unless otherwise noticed, all differential gene expression analyses for this paper used the Wilcoxon test that is powered by FindAllMarkers and FindMarkers functions of Seurat. The FindSpatialNeighborhood analysis was conducted on dermal cells only under default settings. Celltrek, CytoSPACE, Seurat, and Tangram were applied to our in-house SC and ST human skin datasets with the same settings used for benchmarking. Default settings for FindSpatialNeighborhood analysis were also used for Celltrek, CytoSPACE, Seurat, and Tangram predicted human skin dermal results, with minor customization for each method to accommodate their output data structures.

### SPOTlight deconvolution and spatial niche analysis

R package SPOTlight [13] V1.4.1 was applied under the default setting (hvg = 2000, weight_id = "avg_log2FC"). The spatial niche analysis was inspired by the Seurat V5 *BuildNicheAssay* function, while the code was customized to use SPOTlight results as the input with niches.k = 4 and neighbors.k = 30.

### Mouse kidney data acquisition and analysis

We used previously published mouse kidney scRNA-seq data [22] and ST data (10X Genomics Mouse Kidney Section Coronal, spaceranger-1.1.0 processed). scHolography reconstruction used the default settings. Cell type annotation from the original study was adopted. Celltrek, CytoSPACE, Seurat, and Tangram analyses were conducted in the same setting as described. Default settings for FindSpatialNeighborhood analysis were used for PT cells. The Wilcoxon test of FindAllMarkers was used to find markers for each spatial neighborhood.

### Human cSCC data acquisition and analysis

The filtered gene count matrices of the human cSCC 3′ scRNA-seq data were downloaded from GEO (GSE144240), and the cell types were annotated based on the level 2 cell types from the original study [21]. Data were subsetted to keep only Patient 6 data. The keratinocyte cluster without specific keratinocyte state annotations and the multiplet cluster were excluded from downstream processing. The human cSCC ST data was also downloaded from GEO (GSE144240). Only Patient 6 data were processed. The analysis and visualization were handled by automated processing and integration steps of scHolography workflows built upon Seurat (SCTransform normalization, nPCtoUse = 32, FindCluster.resolution = 0.5). We used the cell type annotation from the original study. scHolography prediction of cSCC scRNA-seq data was performed using Patient 6 replicate 1 ST data as the reference. Default settings for FindSpatialNeighborhood analysis were used for tumor KCs (TSK, Tumor KC Basal, Tumor KC Cycling, and Tumor KC Diff). With scHolography results, each non-tumor KC cell type was separated into two groups: (1) the proximal group, containing cells belonging to SMNs of any tumor KCs; (2) the distal group, containing cells not belonging to tumor KC SMNs. Along with original-publication annotated tumor KCs, CellChat was applied under default settings.

### Supplementary Information

Additional file 1: Supplementary figures 1 to 11.

Additional file 2: Supplementary tables 1 to 4.

Additional file 3: Peer review history

## Data Availability

The R package scHolography, tutorials, and scripts used for this manuscript are publicly available on GitHub (https://github.com/YiLab-SC/scHolography) under the GPL-3 license [62]. The code for the analysis of the data presented in this paper can be found on Zenodo [63]. The mouse hippocampus scRNA-seq data is from the Gene Expression Omnibus (GEO) under accession number GSE116470 [64]. The mouse cortex MERFISH data is from the Brain Image Library with 10.35077/g.21 [65]. The human foreskin scRNA-seq and 10X Visium data are in-house generated and deposited under GEO accession number GSE220573 [66]. The mouse kidney data is from GEO under GSE220573 [67]. The human SCC data is from GEO under GSE144240 [68].

## References

[1] Li B, Zhang W, Guo C, Xu H, Li L, Fang M (2022). Benchmarking spatial and single-cell transcriptomics integration methods for transcript distribution prediction and cell type deconvolution. Nat Methods.

[2] Longo SK, Guo MG, Ji AL, Khavari PA (2021). Integrating single-cell and spatial transcriptomics to elucidate intercellular tissue dynamics. Nat Rev Genet.

[3] Palla G, Fischer DS, Regev A, Theis FJ (2022). Spatial components of molecular tissue biology. Nat Biotechnol.

[4] Tang F, Barbacioru C, Wang Y, Nordman E, Lee C, Xu N (2009). mRNA-Seq whole-transcriptome analysis of a single cell. Nat Methods.

[5] Ke R, Mignardi M, Pacureanu A, Svedlund J, Botling J, Wählby C (2013). In situ sequencing for RNA analysis in preserved tissue and cells. Nat Methods.

[6] Lee JH, Daugharthy ER, Scheiman J, Kalhor R, Yang JL, Ferrante TC (2014). Highly multiplexed subcellular RNA sequencing in situ. Science.

[7] Eng CHL, Lawson M, Zhu Q, Dries R, Koulena N, Takei Y (2019). Transcriptome-scale super-resolved imaging in tissues by RNA seqFISH+. Nature..

[8] Chen KH, Boettiger AN, Moffitt JR, Wang S, Zhuang X (2015). Spatially resolved, highly multiplexed RNA profiling in single cells. Science..

[9] Chen A, Liao S, Cheng M, Ma K, Wu L, Lai Y (2022). Spatiotemporal transcriptomic atlas of mouse organogenesis using DNA nanoball-patterned arrays. Cell.

[10] Rodriques SG, Stickels RR, Goeva A, Martin CA, Murray E, Vanderburg CR (2019). Slide-seq: a scalable technology for measuring genome-wide expression at high spatial resolution. Science.

[11] Vickovic S, Eraslan G, Salmén F, Klughammer J, Stenbeck L, Schapiro D (2019). High-definition spatial transcriptomics for in situ tissue profiling. Nat Methods.

[12] Anderson AC, Yanai I, Yates LR, Wang L, Swarbrick A, Sorger P (2022). Spatial transcriptomics. Cancer Cell.

[13] Elosua-Bayes M, Nieto P, Mereu E, Gut I, Heyn H (2021). SPOTlight: seeded NMF regression to deconvolute spatial transcriptomics spots with single-cell transcriptomes. Nucleic Acids Res.

[14] Dong R, Yuan G-C (2021). SpatialDWLS: accurate deconvolution of spatial transcriptomic data. Genome Biol.

[15] Miller BF, Huang F, Atta L, Sahoo A, Fan J (2022). Reference-free cell type deconvolution of multi-cellular pixel-resolution spatially resolved transcriptomics data. Nat Commun.

[16] Cable DM, Murray E, Zou LS, Goeva A, Macosko EZ, Chen F (2022). Robust decomposition of cell type mixtures in spatial transcriptomics. Nat Biotechnol.

[17] Stuart T, Butler A, Hoffman P, Hafemeister C, Papalexi E, Mauck WM (2019). Comprehensive integration of single-cell data. Cell.

[18] Biancalani T, Scalia G, Buffoni L, Avasthi R, Lu Z, Sanger A (2021). Deep learning and alignment of spatially resolved single-cell transcriptomes with Tangram. Nat Methods.

[19] Vahid MR, Brown EL, Steen CB, Zhang W, Jeon HS, Kang M, et al. High-resolution alignment of single-cell and spatial transcriptomes with CytoSPACE. Nat Biotechnol. 2023;41:1543–8. 10.1038/s41587-023-01697-9 PMC10635828 36879008

[20] Wei R, He S, Bai S, Sei E, Hu M, Thompson A (2022). Spatial charting of single-cell transcriptomes in tissues. Nat Biotechnol.

[21] Ji AL, Rubin AJ, Thrane K, Jiang S, Reynolds DL, Meyers RM (2020). Multimodal analysis of composition and spatial architecture in human squamous cell carcinoma. Cell.

[22] Park J, Shrestha R, Qiu C, Kondo A, Huang S, Werth M (2018). Single-cell transcriptomics of the mouse kidney reveals potential cellular targets of kidney disease. Science.

[23] Wagner A, Regev A, Yosef N (2016). Revealing the vectors of cellular identity with single-cell genomics. Nat Biotechnol.

[24] Saunders A, Macosko EZ, Wysoker A, Goldman M, Krienen FM, de Rivera H (2018). Molecular diversity and specializations among the cells of the adult mouse brain. Cell.

[25] Zhang M, Eichhorn SW, Zingg B, Yao Z, Cotter K, Zeng H (2021). Spatially resolved cell atlas of the mouse primary motor cortex by MERFISH. Nature.

[26] Zeira R, Land M, Strzalkowski A, Raphael BJ (2022). Alignment and integration of spatial transcriptomics data. Nat Methods.

[27] Driskell RR, Watt FM (2015). Understanding fibroblast heterogeneity in the skin. Trends Cell Biol.

[28] Fuchs E (2016). Epithelial skin biology: three decades of developmental biology, a hundred questions answered and a thousand new ones to address. Curr Top Dev Biol.

[29] Hao Y, Hao S, Andersen-Nissen E, Mauck WM, Zheng S, Butler A (2021). Integrated analysis of multimodal single-cell data. Cell.

[30] Philippeos C, Telerman SB, Oulès B, Pisco AO, Shaw TJ, Elgueta R (2018). Spatial and single-cell transcriptional profiling identifies functionally distinct human dermal fibroblast subpopulations. J Invest Dermatol.

[31] Driskell RR, Lichtenberger BM, Hoste E, Kretzschmar K, Simons BD, Charalambous M (2013). Distinct fibroblast lineages determine dermal architecture in skin development and repair. Nature.

[32] Hsu Y-C, Li L, Fuchs E (2014). Emerging interactions between skin stem cells and their niches. Nat Med.

[33] Wang S, Drummond ML, Guerrero-Juarez CF, Tarapore E, MacLean AL, Stabell AR (2020). Single cell transcriptomics of human epidermis identifies basal stem cell transition states. Nat Commun.

[34] Jassal B, Matthews L, Viteri G, Gong C, Lorente P, Fabregat A (2020). The reactome pathway knowledgebase. Nucleic Acids Res.

[35] Blanpain C, Lowry WE, Pasolli HA, Fuchs E (2006). Canonical notch signaling functions as a commitment switch in the epidermal lineage. Genes Dev.

[36] Moriyama M, Durham AD, Moriyama H, Hasegawa K, Nishikawa S, Radtke F (2008). Multiple roles of Notch signaling in the regulation of epidermal development. Dev Cell.

[37] Jin S, Guerrero-Juarez CF, Zhang L, Chang I, Ramos R, Kuan C-H (2021). Inference and analysis of cell-cell communication using Cell Chat. Nat Commun.

[38] Broussard JA, Koetsier JL, Hegazy M, Green KJ (2021). Desmosomes polarize and integrate chemical and mechanical signaling to govern epidermal tissue form and function. Curr Biol.

[39] Hegazy M, Perl AL, Svoboda SA, Green KJ (2022). Desmosomal cadherins in health and disease. Annu Rev Pathol.

[40] Hao Y, Stuart T, Kowalski MH, Choudhary S, Hoffman P, Hartman A (2024). Dictionary learning for integrative, multimodal and scalable single-cell analysis. Nat Biotechnol.

[41] Ransick A, Lindström NO, Liu J, Zhu Q, Guo J-J, Alvarado GF (2019). Single-cell profiling reveals sex, lineage, and regional diversity in the mouse kidney. Dev Cell.

[42] Sánchez-Danés A, Blanpain C (2018). Deciphering the cells of origin of squamous cell carcinomas. Nat Rev Cancer.

[43] Gavish A, Tyler M, Greenwald AC, Hoefflin R, Simkin D, Tschernichovsky R (2023). Hallmarks of transcriptional intratumour heterogeneity across a thousand tumours. Nature.

[44] Barkley D, Moncada R, Pour M, Liberman DA, Dryg I, Werba G (2022). Cancer cell states recur across tumor types and form specific interactions with the tumor microenvironment. Nat Genet.

[45] Liberzon A, Birger C, Thorvaldsdóttir H, Ghandi M, Mesirov JP, Tamayo P (2015). The molecular signatures database hallmark gene set collection. Cell Syst.

[46] Faget J, Bendriss-Vermare N, Gobert M, Durand I, Olive D, Biota C (2012). ICOS-ligand expression on plasmacytoid dendritic cells supports breast cancer progression by promoting the accumulation of immunosuppressive CD4+ T cells. Cancer Res.

[47] Han N, Zhang Z, Liu S, Ow A, Ruan M, Yang W (2017). Increased tumor-infiltrating plasmacytoid dendritic cells predicts poor prognosis in oral squamous cell carcinoma. Arch Oral Biol.

[48] Hartmann E, Wollenberg B, Rothenfusser S, Wagner M, Wellisch D, Mack B (2003). Identification and functional analysis of tumor-infiltrating plasmacytoid dendritic cells in head and neck cancer. Cancer Res.

[49] Sancho D, Joffre OP, Keller AM, Rogers NC, Martínez D, Hernanz-Falcón P (2009). Identification of a dendritic cell receptor that couples sensing of necrosis to immunity. Nature.

[50] Kvedaraite E, Ginhoux F (2022). Human dendritic cells in cancer. Sci Immunol..

[51] Hildner K, Edelson BT, Purtha WE, Diamond M, Matsushita H, Kohyama M (2008). Batf3 deficiency reveals a critical role for CD8alpha+ dendritic cells in cytotoxic T cell immunity. Science.

[52] Chollet F, et al. R interface to Keras. GitHub. 2017. https://github.com/rstudio/keras3.

[53] Tilly J, Janetos N. Matching algorithms in R and C++. GitHub. 2015. https://github.com/jtilly/matchingR.

[54] Csardi G, Nepusz T (2006). The igraph software package for complex network research. InterJournal Complex Syst.

[55] Tasic B, Menon V, Nguyen TN, Kim TK, Jarsky T, Yao Z, et al. Adult mouse cortical cell taxonomy revealed by single cell transcriptomics. Nat Neurosci. 2016;19:335–46. 10.1038/nn.4216 PMC4985242 26727548

[56] Stickels RR, Murray E, Kumar P, Li J, Marshall JL, Di Bella DJ (2021). Highly sensitive spatial transcriptomics at near-cellular resolution with Slide-seqV2. Nat Biotechnol.

[57] Moffitt JR, Bambah-Mukku D, Eichhorn SW, Vaughn E, Shekhar K, Perez JD (2018). Molecular, spatial, and functional single-cell profiling of the hypothalamic preoptic region. Science..

[58] Korsunsky I, Millard N, Fan J, Slowikowski K, Zhang F, Wei K (2019). Fast, sensitive and accurate integration of single-cell data with Harmony. Nat Methods.

[59] Welch JD, Kozareva V, Ferreira A, Vanderburg C, Martin C, Macosko EZ (2019). Single-cell multi-omic integration compares and contrasts features of brain cell identity. Cell.

[60] Haghverdi L, Lun ATL, Morgan MD, Marioni JC (2018). Batch effects in single-cell RNA-sequencing data are corrected by matching mutual nearest neighbors. Nat Biotechnol.

[61] Hafemeister C, Satija R (2019). Normalization and variance stabilization of single-cell RNA-seq data using regularized negative binomial regression. Genome Biol..

[62] Fu YC. scHolography: a computational method for single-cell spatial neighborhood reconstruction and analysis. 2024. GitHub. https://github.com/YiLab-SC/scHolography. 10.1186/s13059-024-03299-3 PMC11197379 38915088

[63] Fu YC. scHolography: a computational method for single-cell spatial neighborhood reconstruction and analysis. 2024. Zenodo. https://zenodo.org/records/11218131. 10.1186/s13059-024-03299-3 PMC11197379 38915088

[64] Saunders A, Macosko EZ, Wysoker A, Goldman M et al. A single-cell atlas of cell types, states, and other transcriptional patterns from nine regions of the adult mouse brain. Datasets. Gene Expression Omnibus. 2018. https://www.ncbi.nlm.nih.gov/geo/query/acc.cgi?acc=GSE116470.

[65] Zhuang X, Zhang M. A molecularly defined and spatially resolved cell atlas of the mouse primary motor cortex. Datasets. Brain Image Library. 2020. 10.35077/g.21.

[66] Fu Y, Das A, Wang D, Braun R, Rui Yi. Reconstruction of 3-dimensional tissue organization at the single-cell resolution. Datasets. Gene Expression Omnibus. 2023. https://www.ncbi.nlm.nih.gov/geo/query/acc.cgi?acc=GSE220573.

[67] Park J, Shrestha R, Qiu C, Kondo A, Huang S, Werth M, Li M, Barasch J, Suszták K. Comprehensive single cell RNAseq analysis of the kidney reveals novel cell types and unexpected cell plasticity. Datasets. Gene Expression Omnibus. 2018. https://www.ncbi.nlm.nih.gov/geo/query/acc.cgi?acc=GSE107585.

[68] Ji AL, Rubin AJ, Thrane K, Jiang S et al. Multimodal analysis of composition and spatial architecture in human squamous cell carcinoma. Datasets. Gene Expression Omnibus. 2020. https://www.ncbi.nlm.nih.gov/geo/query/acc.cgi?acc=GSE144240. 10.1016/j.cell.2020.05.039 PMC7391009 32579974

