# Supplementary material for: scHolography: a computational method for single-cell spatial neighborhood reconstruction and analysis
Source: Genome Biol. 2024 Jun 24;25:164. doi: 10.1186/s13059-024-03299-3 (PMC11197379; doi:10.1186/s13059-024-03299-3)
Supplement: Supplementary file 1 — Additional file 1: Supplementary figures 1 to 11. [file 13059_2024_3299_MOESM1_ESM.pdf]

Figure S1

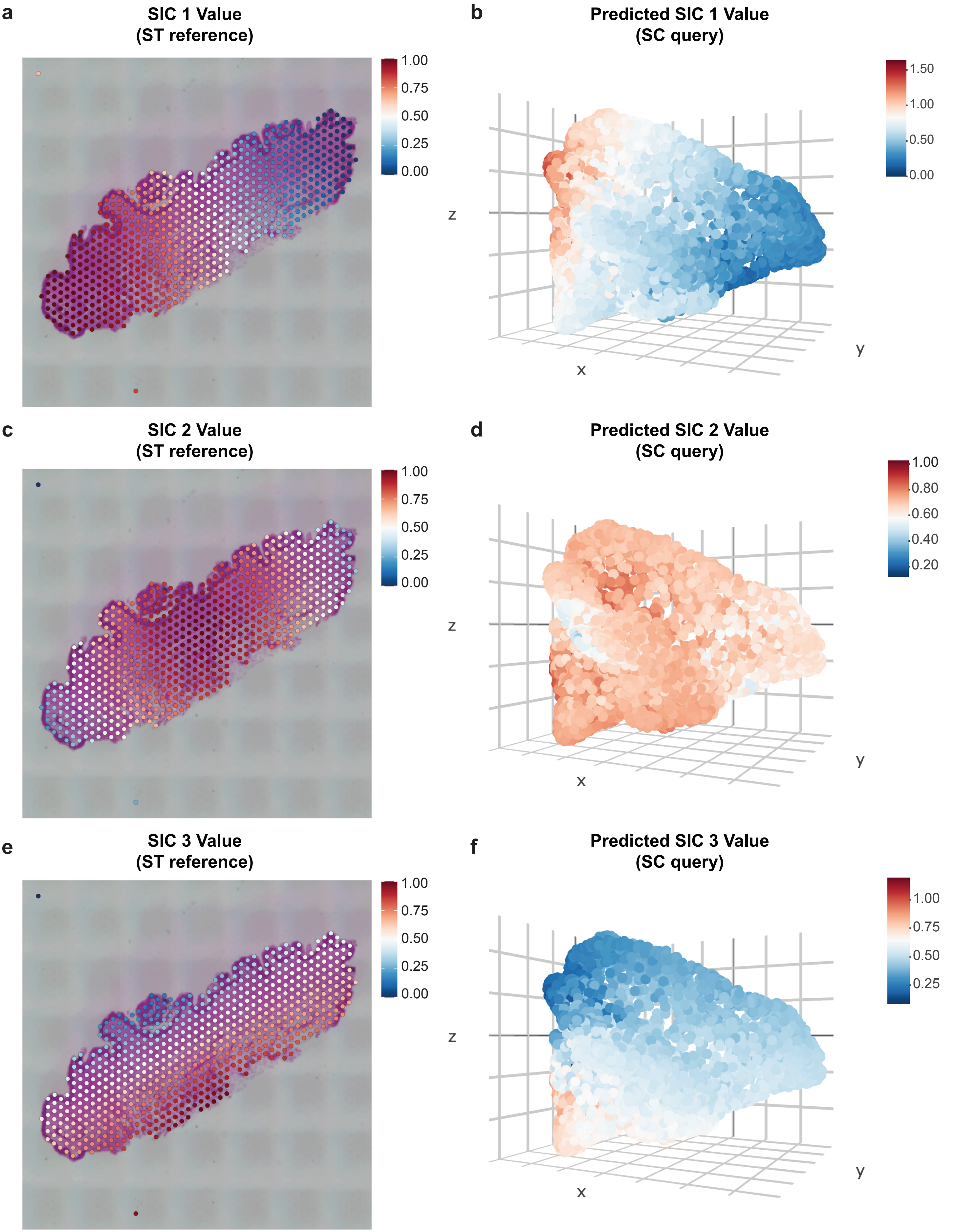
a
SIC 1 Value
(ST reference)
1.00
0.75
0.50
0.25
0.00
b
Predicted SIC 1 Value
(SC query)
1.50
1.00
0.50
0.00
z
x
y
c
SIC 2 Value
(ST reference)
1.00
0.75
0.50
0.25
0.00
d
Predicted SIC 2 Value
(SC query)
1.00
0.80
0.60
0.40
0.20
z
x
y
e
SIC 3 Value
(ST reference)
1.00
0.75
0.50
0.25
0.00
f
Predicted SIC 3 Value
(SC query)
1.00
0.75
0.50
0.25
z
x
y

Figure S1. Spatial relevance of SICs. Top three SIC values in human skin ST data on the spatial image (left panels) and predicted top three SIC values in 3D visualization of scHolography reconstruction of human skin SC data (right panels). **a-b,** SIC 1 values in ST and predicted SIC 1 values in SC data. **c-d,** SIC 2 values in ST and predicted SIC 2 values in SC data. **e-f,** SIC 3 values in ST and predicted SIC 3 values in SC data.

Figure S2

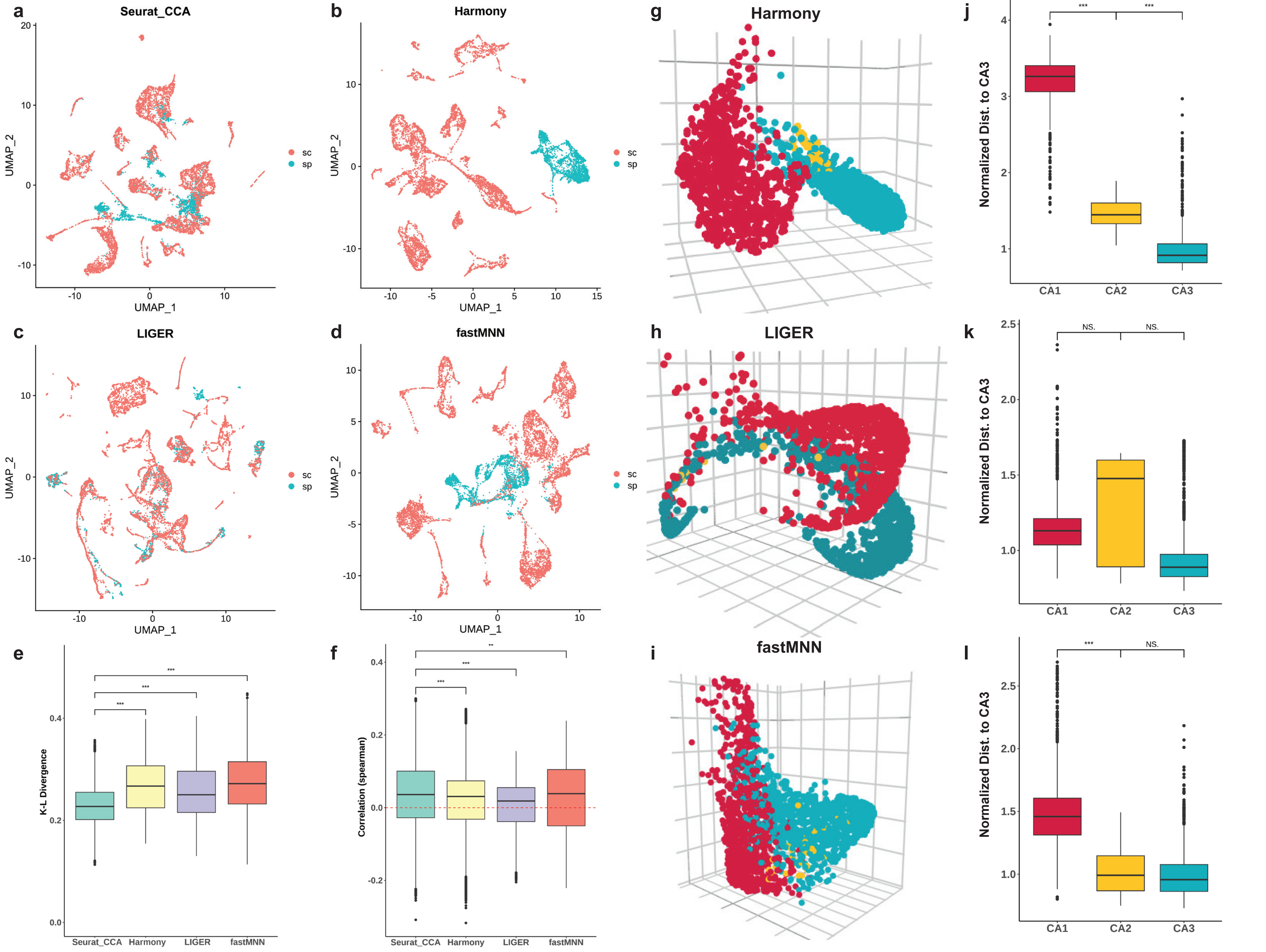
a
Seurat_CCA
b
Harmony
g
Harmony
j
c
LIGER
d
fastMNN
h
LIGER
k
e
f
i
fastMNN
l
UMAP_1
UMAP_2
sc
sp
Normalized Dist. to CA3
CA1
CA2
CA3
***
NS.
K-L Divergence
Correlation (spearman)
Seurat_CCA
Harmony
LIGER
fastMNN
**

Figure S2. a, UMAP plot of Seurat CCA integration of scRNA-seq and ST data. **b,** UMAP plot of Harmony integration of scRNA-seq and ST data. **c,** UMAP plot of LIGER integration of scRNA-seq and ST data. **d,** UMAP plot of fastMNN integration of scRNA-seq and ST data. **e,** KL-divergence of scHolography predictions powered by different integration methods for simulated mouse hippocampus data as ground truth. Two-sided Wilcoxon tests are performed. **f,** Spearman correlation between scHolography cell-cell SMN distance powered by different integration methods and simulated mouse hippocampus cell-cell distance. Two-sided Wilcoxon tests are performed. **g-i,** Visualization of Harmony, LIGER, and fastMNN powered scHolography prediction results. **j-l,** Comparison of Harmony, LIGER, and fastMNN powered scHolography reconstruction results for CA1, CA2, and CA3 cell distance to CA3 cells. Cell distances were normalized for each method by the mean distance between CA3 cells and CA3 cells. Two-sided Wilcoxon tests are performed.

Figure S3

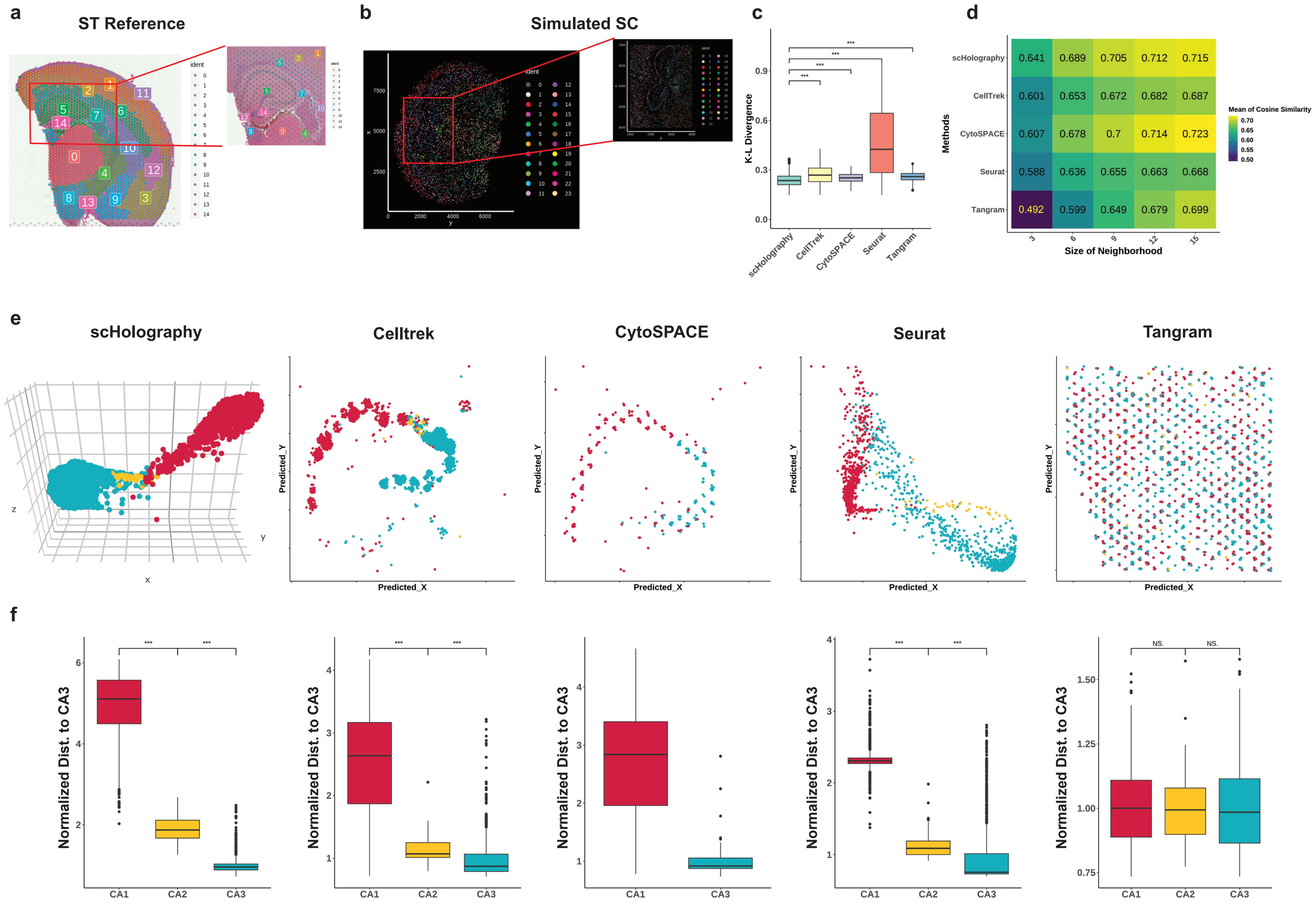

a
ST Reference
b
Simulated SC
c
K-L Divergence
scHolography
CellTrek
CytoSPACE
Seurat
Tangram
d
Methods
Size of Neighborhood
Mean of Cosine Similarity
e
scHolography
Celltrek
CytoSPACE
Seurat
Tangram
Predicted_X
Predicted_Y
f
Normalized Dist. to CA3
CA1
CA2
CA3

Figure S3. a, Illustration of 10X Visium mouse hippocampus ST reference region. **b,** Illustration of mouse hippocampus simulated SC query region. **c,** KL-divergence of spatial cell charting method predictions for simulated mouse hippocampus data as ground truth. Two-sided Wilcoxon tests are performed. **d,** Heatmap for the mean of cosine similarity between method-predicted spatial neighborhood accumulated expression and simulated mouse hippocampus spatial neighborhood accumulated expression. The size of the neighborhood varies from 3 to 15 cells. **e,** Visualization of scHolography, Celltrek, CytoSPACE, Seurat, and Tangram single-cell spatial charting results of a mouse hippocampus data. **f,** Comparison of scHolography, Celltrek, CytoSPACE, Seurat, and Tangram results for predicted CA1, CA2, and CA3 cell distance to CA3 cells. Cell distances were normalized for each method by the mean distance between CA3 cells and CA3 cells. Two-sided Wilcoxon tests are performed.

Figure S4

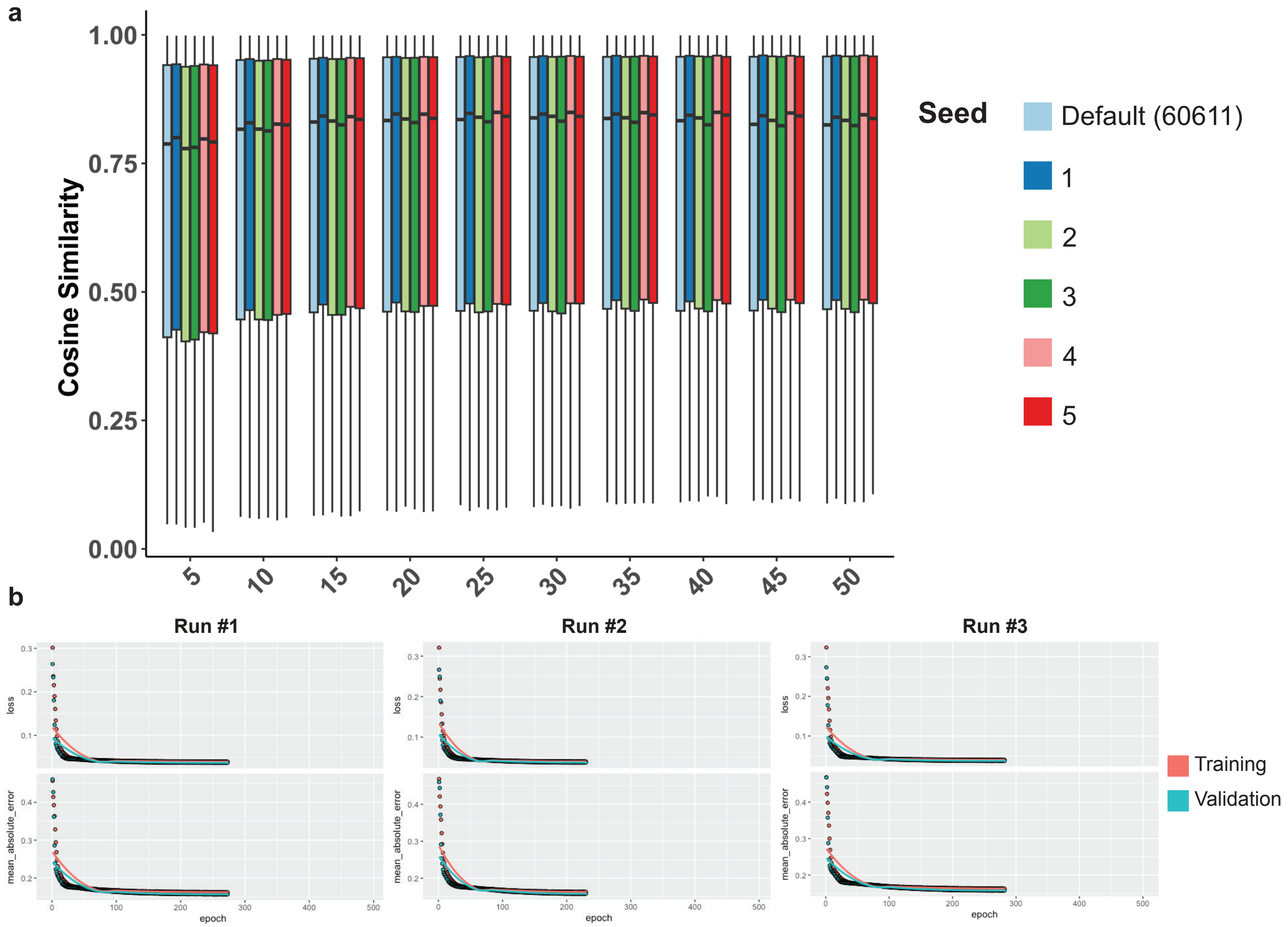
a
Cosine Similarity
1.00
0.75
0.50
0.25
0.00
5
10
15
20
25
30
35
40
45
50
Seed
Default (60611)
1
2
3
4
5
b
Run #1
Run #2
Run #3
loss
mean_absolute_error
epoch
Training
Validation

Figure S4. a, Boxplot for the mean of cosine similarity between scHolography-predicted spatial neighborhood accumulated expression under different initialization seeds and simulated mouse hippocampus spatial neighborhood accumulated expression. The size of the neighborhood varies from 5 to 50 cells. **b,** Training and validation loss and mean absolute error curves for the first three runs of 30 repeat runs of neural network training.

Figure S5

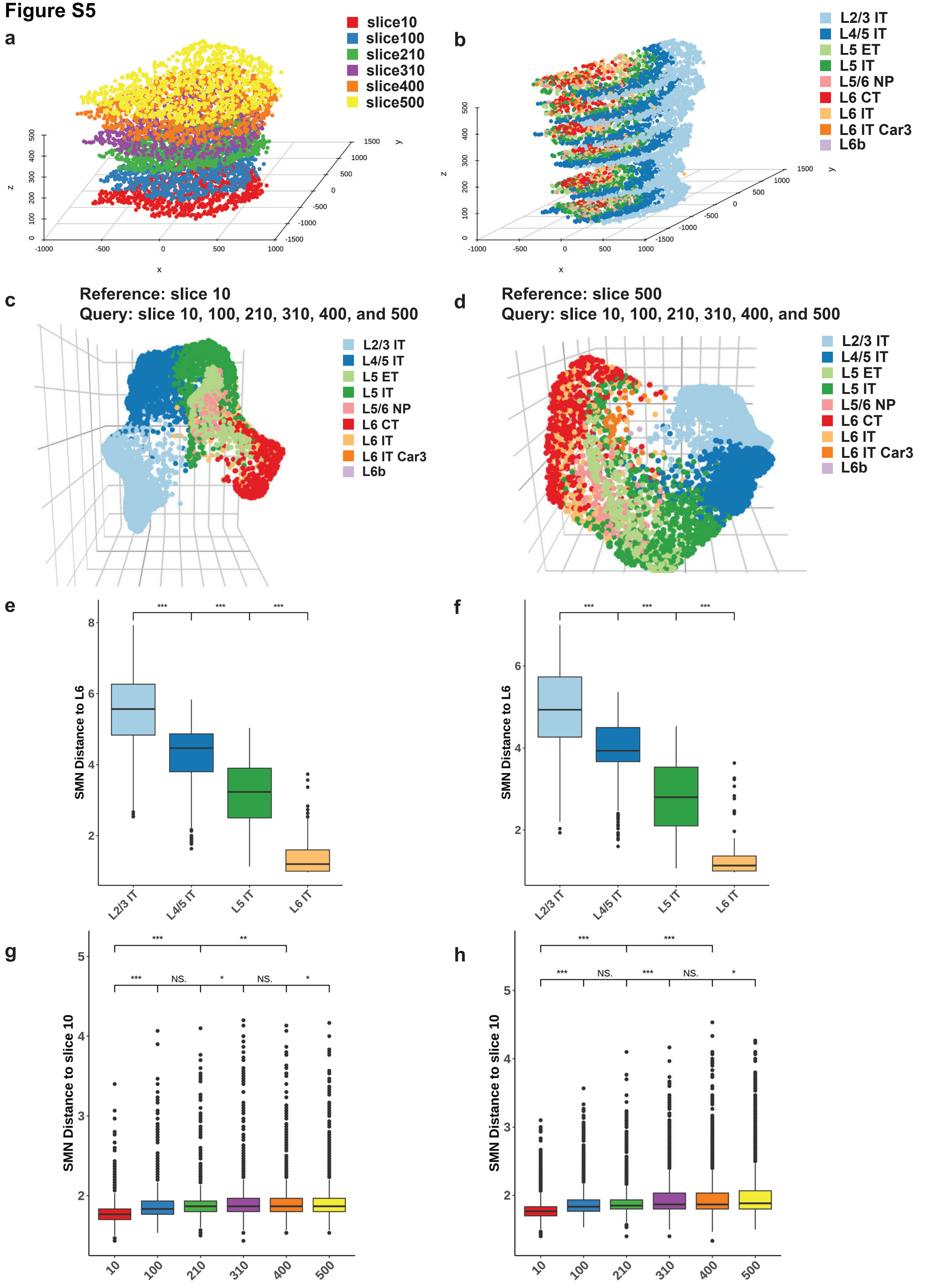
a
slice10
slice100
slice210
slice310
slice400
slice500
b
L2/3 IT
L4/5 IT
L5 ET
L5 IT
L5/6 NP
L6 CT
L6 IT
L6 IT Car3
L6b
c
Reference: slice 10
Query: slice 10, 100, 210, 310, 400, and 500
d
Reference: slice 500
Query: slice 10, 100, 210, 310, 400, and 500
e
SMN Distance to L6
f
SMN Distance to L6
L2/3 IT
L4/5 IT
L5 IT
L6 IT
g
SMN Distance to slice 10
h
SMN Distance to slice 10
NS.
10
100
210
310
400
500

Figure S5. a, Stacked-2D plot of Merfish mouse cortex sample 1 (slice 10, 100, 210, 310, 400, and 500) colored by the slice. b, Stacked-2D plot of Merfish mouse cortex sample 1 (slice 10, 100, 210, 310, 400, and 500) colored by cell types. **c-d,** 3D visualization of Merfish sample1 scHolography reconstruction result. **c,** use slice 10 as the reference; **d,** use slice 500 as the reference. **e-f,** SMN distances from L2/3 IT, L4/5 IT, L5 IT, L6 IT to L6 IT in Merfish sample1 scHolography prediction. Two-sided Wilcoxon tests are performed. **e,** use slice 10 as the reference; **f,** use slice 500 as the reference. **g-h,** SMN distances from slice 10, 100, 210, 310, 400, and 500 to slice 10 in Merfish sample 1 scHolography prediction. Two-sided Wilcoxon tests are performed. **g,** use slice 10 as the reference; h, use slice 500 as the reference.

Figure S6

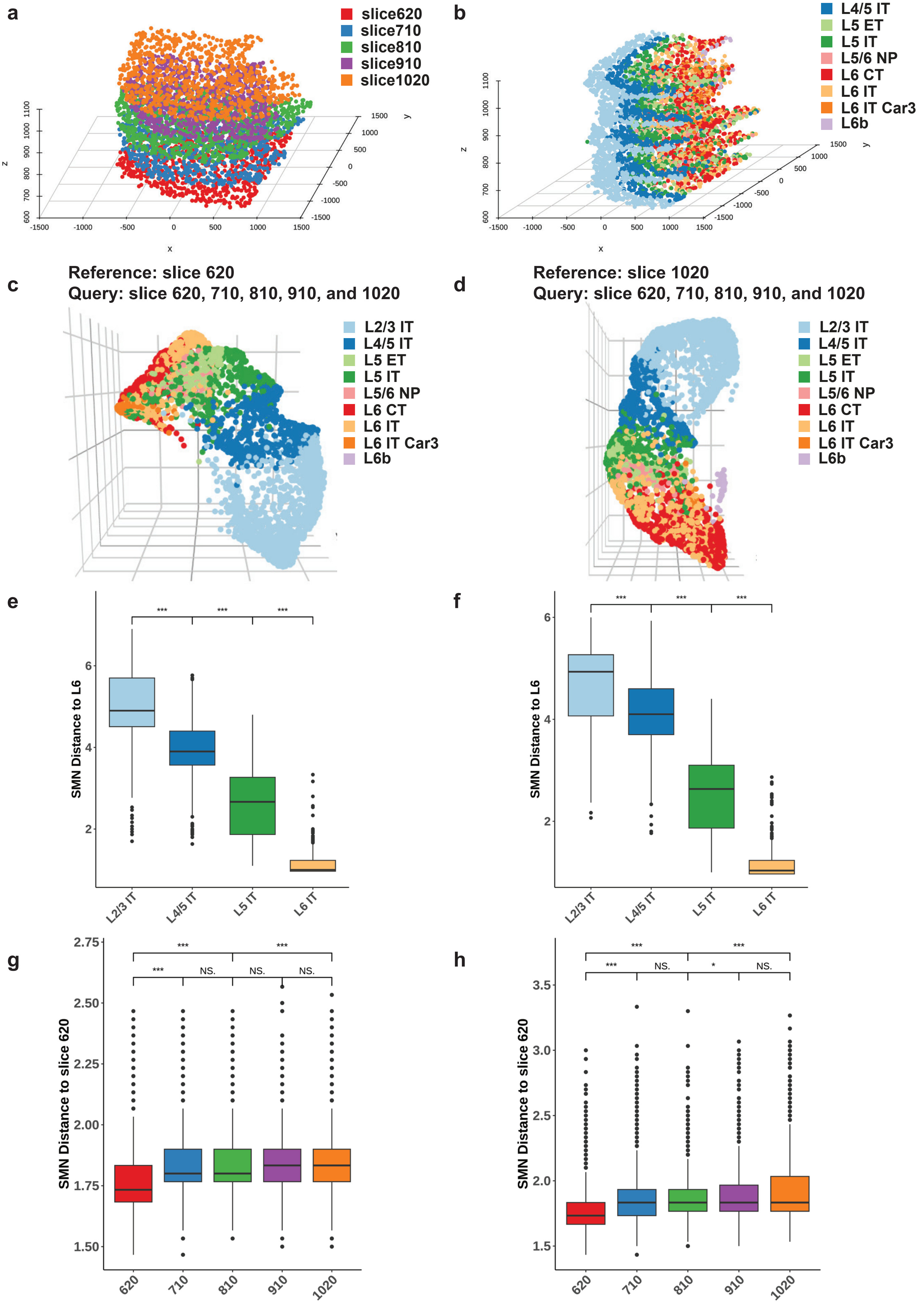
a
slice620
slice710
slice810
slice910
slice1020
b
L2/3 IT
L4/5 IT
L5 ET
L5 IT
L5/6 NP
L6 CT
L6 IT
L6 IT Car3
L6b
c
Reference: slice 620
Query: slice 620, 710, 810, 910, and 1020
d
Reference: slice 1020
Query: slice 620, 710, 810, 910, and 1020
e
SMN Distance to L6
f
SMN Distance to L6
L2/3 IT
L4/5 IT
L5 IT
L6 IT
g
SMN Distance to slice 620
h
SMN Distance to slice 620
620
710
810
910
1020
***
NS.
*

Figure S6. a, Stacked-2D plot of Merfish mouse cortex sample 2 (620, 710, 810, 910, and 1020) colored by the slice. **b,** Stacked-2D plot of Merfish mouse cortex sample 1 (620, 710, 810, 910, and 1020) colored by cell types. **c-d,** 3D visualization of Merfish sample2 scHolography reconstruction result. **c,** use slice 620 as the reference; **d,** use slice 1020 as the reference. **e-f,** SMN distances from L2/3 IT, L4/5 IT, L5 IT, L6 IT to L6 IT in Merfish sample2 scHolography prediction. Two-sided Wilcoxon tests are performed. **e,** use slice 620 as the reference; **f,** use slice 1020 as the reference. **g-h,** SMN distances from slice 620, 710, 810, 910, and 1020 to slice 620 in Merfish sample 2 scHolography prediction. Two-sided Wilcoxon tests are performed. **g,** use slice 620 as the reference; **h,** use slice 1020 as the reference.

Figure S7

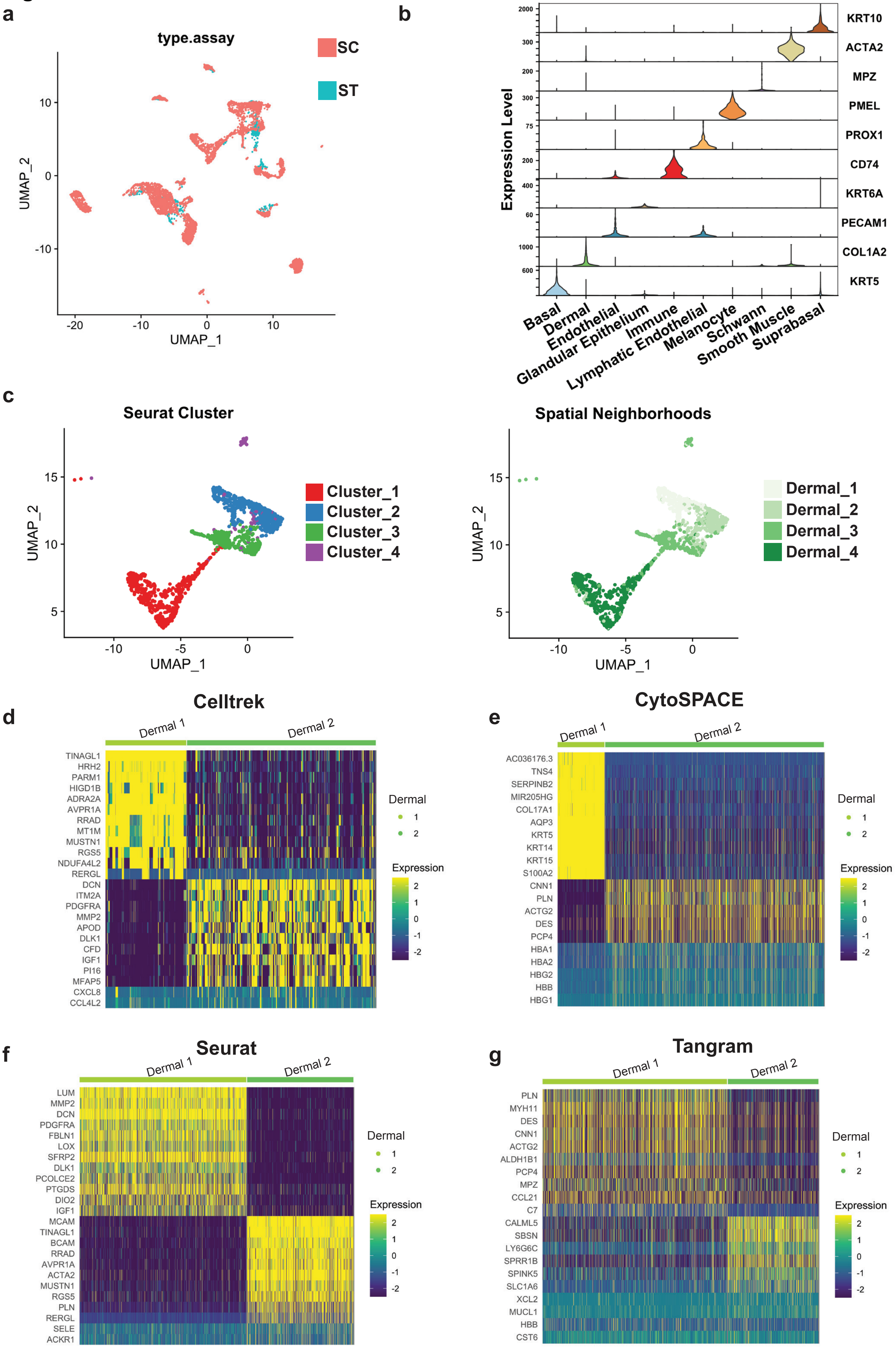
a
type.assay
SC
ST
UMAP_2
UMAP_1
b
Expression Level
KRT10
ACTA2
MPZ
PMEL
PROX1
CD74
KRT6A
PECAM1
COL1A2
KRT5
Basal
Dermal
Endothelial
Glandular Epithelium
Immune
Lymphatic Endothelial
Melanocyte
Schwann
Smooth Muscle
Suprabasal
c
Seurat Cluster
Cluster_1
Cluster_2
Cluster_3
Cluster_4
Spatial Neighborhoods
Dermal_1
Dermal_2
Dermal_3
Dermal_4
d
Celltrek
Dermal 1
Dermal 2
TINAGL1
HRH2
PARM1
HIGD1B
ADRA2A
AVPR1A
RRAD
MT1M
MUSTN1
RGS5
NDUFA4L2
RERGL
DCN
ITM2A
PDGFRA
MMP2
APOD
DLK1
CFD
IGF1
PI16
MFAP5
CXCL8
CCL4L2
Dermal
Expression
e
CytoSPACE
AC036176.3
TNS4
SERPINB2
MIR205HG
COL17A1
AQP3
KRT5
KRT14
KRT15
S100A2
CNN1
PLN
ACTG2
DES
PCP4
HBA1
HBA2
HBG2
HBB
HBG1
f
Seurat
LUM
MMP2
DCN
PDGFRA
FBLN1
LOX
SFRP2
DLK1
PCOLCE2
PTGDS
DIO2
IGF1
MCAM
TINAGL1
BCAM
RRAD
AVPR1A
ACTA2
MUSTN1
RGS5
PLN
RERGL
SELE
ACKR1
g
Tangram
PLN
MYH11
DES
CNN1
ACTG2
ALDH1B1
PCP4
MPZ
CCL21
C7
CALML5
SBSN
LY6G6C
SPRR1B
SPINK5
SLC1A6
XCL2
MUCL1
HBB
CST6

Figure S7. a, Human foreskin SC and ST data integration. **b**, Identification of major cell types in human foreskin samples by cell lineage markers. *KRT10*, suprabasal epithelial cells; *ACTA2*, smooth muscle cells.; *MPZ*, Schwann cells; *PMEL*, melanocytes; *PROX1*, lymphatic endothelial cells; *CD74*, immune cells; *KRT6A*, glandular epithelial cells; *COL1A2*, dermal cells; *PECAM1*, endothelial cells; *KRT5*, basal epithelial cells. **c**, Comparison between transcriptome-based Seurat dermal cell clusters (left) and scHolography identified dermal spatial neighborhoods (right). **d-g**, Heatmaps of top 10 differentially expressed genes for the dermal spatial neighborhood accumulated SMN expression profile. The spatial neighborhoods were identified based on Celltrek, CytoSPACE, Seurat, and Tangram prediction.

Figure S8

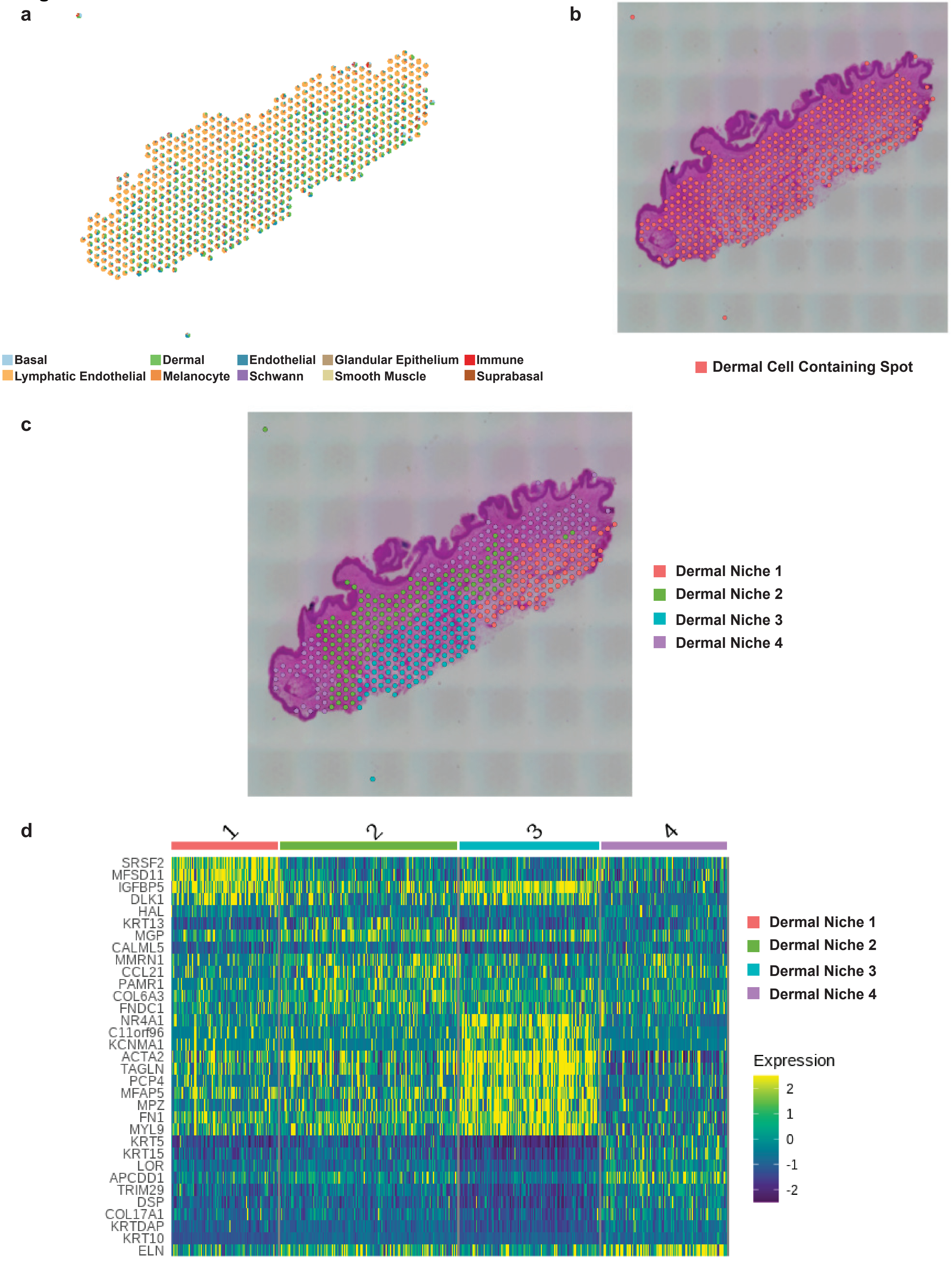
a
b
Basal
Lymphatic Endothelial
Dermal
Melanocyte
Endothelial
Schwann
Glandular Epithelium
Smooth Muscle
Immune
Suprabasal
Dermal Cell Containing Spot
c
Dermal Niche 1
Dermal Niche 2
Dermal Niche 3
Dermal Niche 4
d
1
2
3
4
SRSF2
MFSD11
IGFBP5
DLK1
HAL
KRT13
MGP
CALML5
MMRN1
CCL21
PAMR1
COL6A3
FNDC1
NR4A1
C11orf96
KCNMA1
ACTA2
TAGLN
PCP4
MFAP5
MPZ
FN1
MYL9
KRT5
KRT15
LOR
APCDD1
TRIM29
DSP
COL17A1
KRTDAP
KRT10
ELN
Dermal Niche 1
Dermal Niche 2
Dermal Niche 3
Dermal Niche 4
Expression
2
1
0
-1
-2

Figure S8. a, SPOTlight deconvolution results for human skin 10X Visium data. **b,** Defined dermal cell-containing spatial spots. A spot is defined as a dermal cell-containing spatial spot when its dermal cell composition is inferred to be greater than 0.15 by SPOTlight. **c,** Spatial niche analysis for dermal cell-containing spatial spots. **d,** Heatmap of top DEG for each spatial niche.

Figure S9

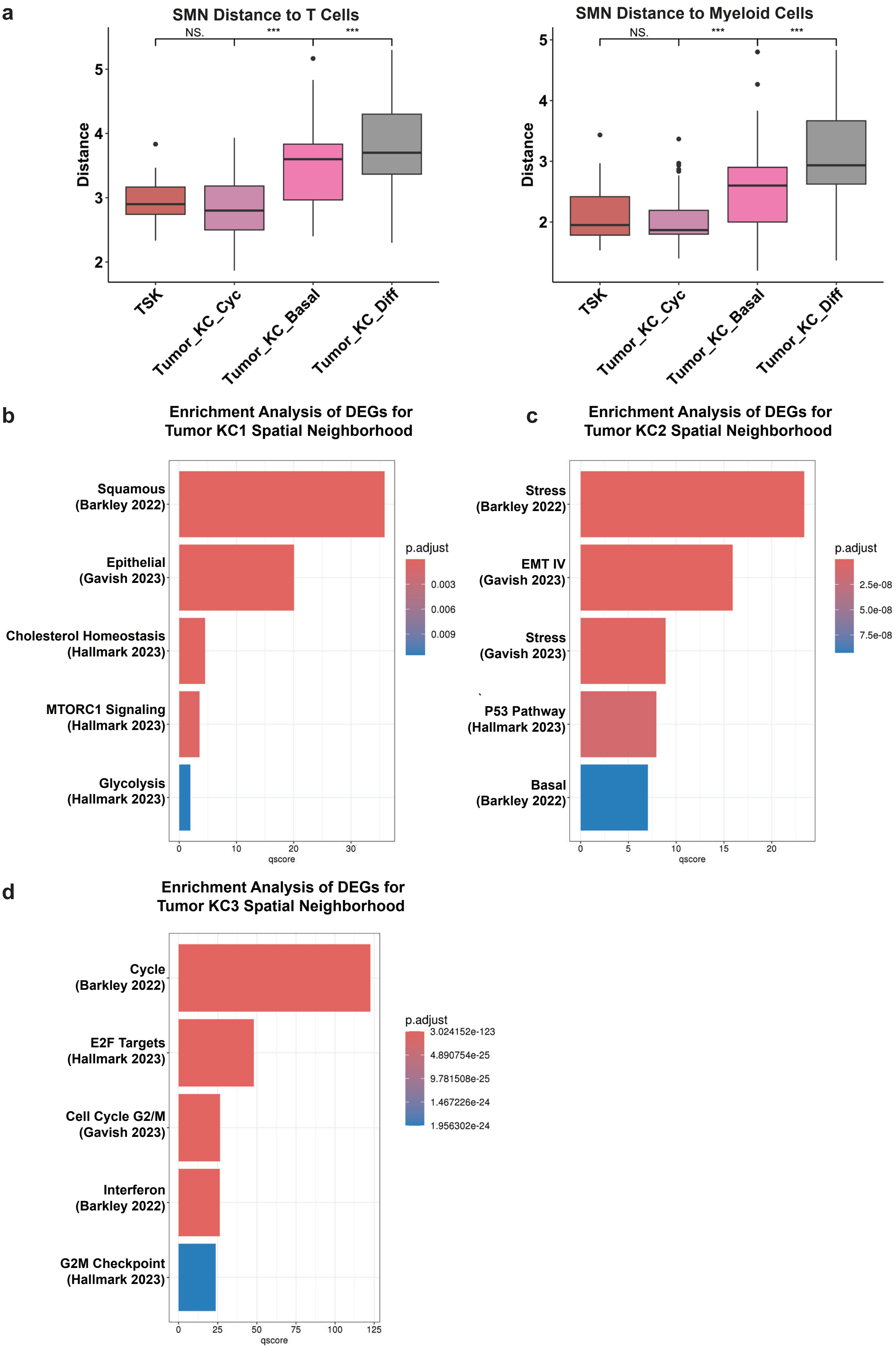
a
SMN Distance to T Cells
NS.
***
***
Distance
5
4
3
2
TSK
Tumor_KC_Cyc
Tumor_KC_Basal
Tumor_KC_Diff
SMN Distance to Myeloid Cells
NS.
***
***
Distance
5
4
3
2
TSK
Tumor_KC_Cyc
Tumor_KC_Basal
Tumor_KC_Diff
b
Enrichment Analysis of DEGs for
Tumor KC1 Spatial Neighborhood
Squamous
(Barkley 2022)
Epithelial
(Gavish 2023)
Cholesterol Homeostasis
(Hallmark 2023)
MTORC1 Signaling
(Hallmark 2023)
Glycolysis
(Hallmark 2023)
0
10
20
30
qscore
p.adjust
0.003
0.006
0.009
c
Enrichment Analysis of DEGs for
Tumor KC2 Spatial Neighborhood
Stress
(Barkley 2022)
EMT IV
(Gavish 2023)
Stress
(Gavish 2023)
P53 Pathway
(Hallmark 2023)
Basal
(Barkley 2022)
0
5
10
15
20
qscore
p.adjust
2.5e-08
5.0e-08
7.5e-08
d
Enrichment Analysis of DEGs for
Tumor KC3 Spatial Neighborhood
Cycle
(Barkley 2022)
E2F Targets
(Hallmark 2023)
Cell Cycle G2/M
(Gavish 2023)
Interferon
(Barkley 2022)
G2M Checkpoint
(Hallmark 2023)
0
25
50
75
100
125
qscore
p.adjust
3.024152e-123
4.890754e-25
9.781508e-25
1.467226e-24
1.956302e-24

Figure S9. a, Violin plot of SMN distance between different Tumor KC cell types and T cells (left). Violin plot of SMN distance between different Tumor KC cell types and myeloid cells (right). **b**-**d**, Enrichment analysis of DEGs (Mann Whitney Wilcoxon test, p_adjusted<0.05) for Tumor KC1 (**b**), Tumor KC2 (**c**), and Tumor KC3 (**d**) accumulated spatial neighborhood expression profiles. Hypergeometric tests were performed for enrichment analysis, and the top 5 term hits were displayed.

Figure S10

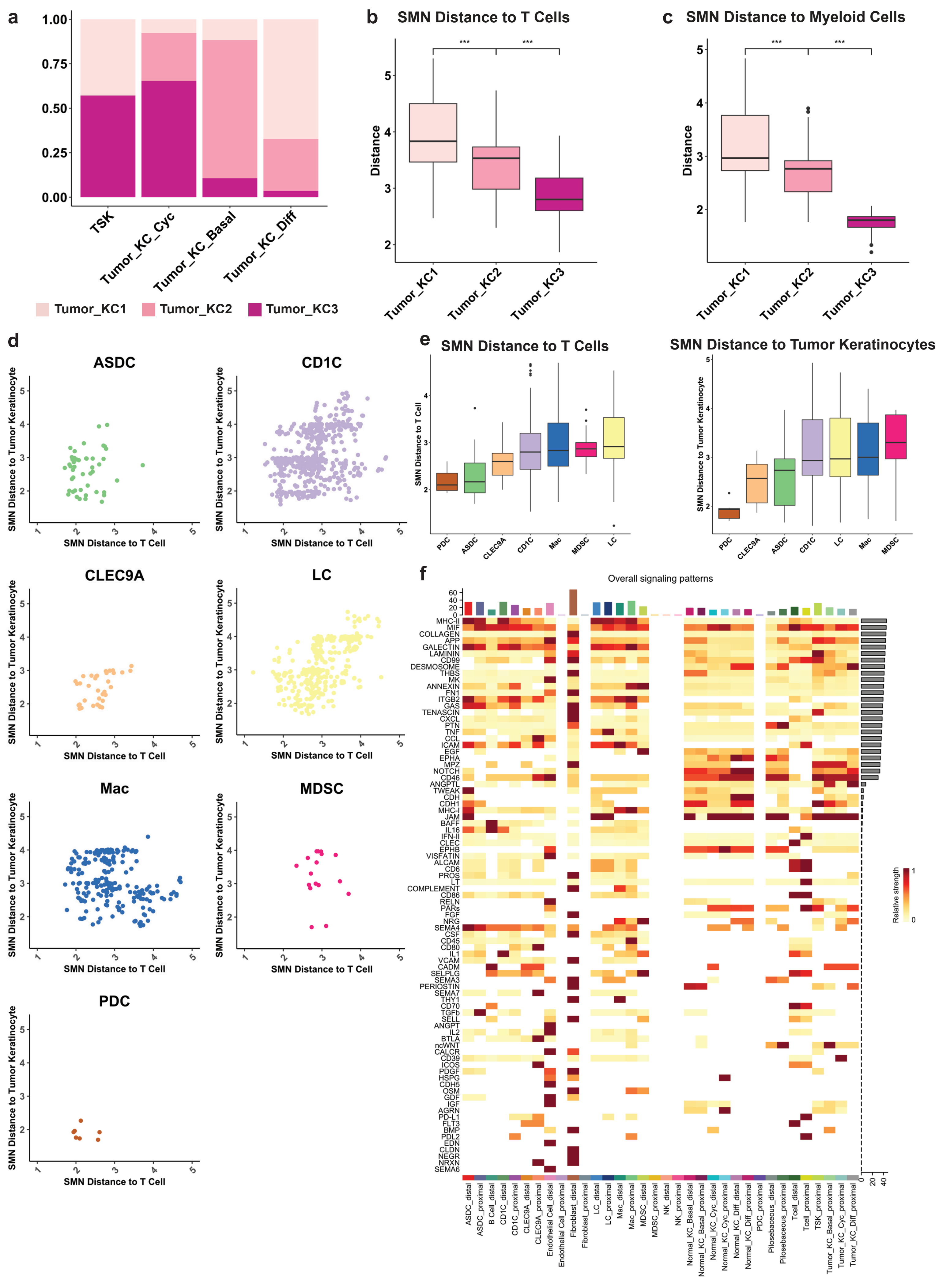

a
Tumor_KC1
Tumor_KC2
Tumor_KC3
TSK
Tumor_KC_Cyc
Tumor_KC_Basal
Tumor_KC_Diff
b
SMN Distance to T Cells
Distance
c
SMN Distance to Myeloid Cells
d
ASDC
CD1C
CLEC9A
LC
Mac
MDSC
PDC
SMN Distance to Tumor Keratinocyte
SMN Distance to T Cell
e
SMN Distance to T Cells
SMN Distance to Tumor Keratinocytes
PDC
ASDC
CLEC9A
CD1C
Mac
MDSC
LC
f
Overall signaling patterns
Relative strength

Figure S10. a, Composition of spatial neighborhood assignment for different Tumor KC cell types. **b**-**c**, Violin plot of SMN distance between different Tumor KC spatial neighborhoods and T cells (**b**). Violin plot of SMN distance between different Tumor KC spatial neighborhoods and myeloid cells (**c**). **d**, SMN distances between myeloid cells and T cells vs. SMN distances between myeloid cells and tumor keratinocyte cells, break down for each myeloid cell type. **e**, Violin plot of SMN distance between different myeloid cell types and T cells (left). Violin plot of SMN distance between different myeloid cell types and tumor keratinocyte cells (right). **f**, Overall signaling strengths inferred by CellChat for different cell types in cSCC. Based on scHolography, each cell type is split into (1) distal and (2) proximal groups. Cells in the distal group are not SMNs of any tumor keratinocyte cells, while cells in the proximal group are SMNs of at least one tumor keratinocyte cell.

Figure S11

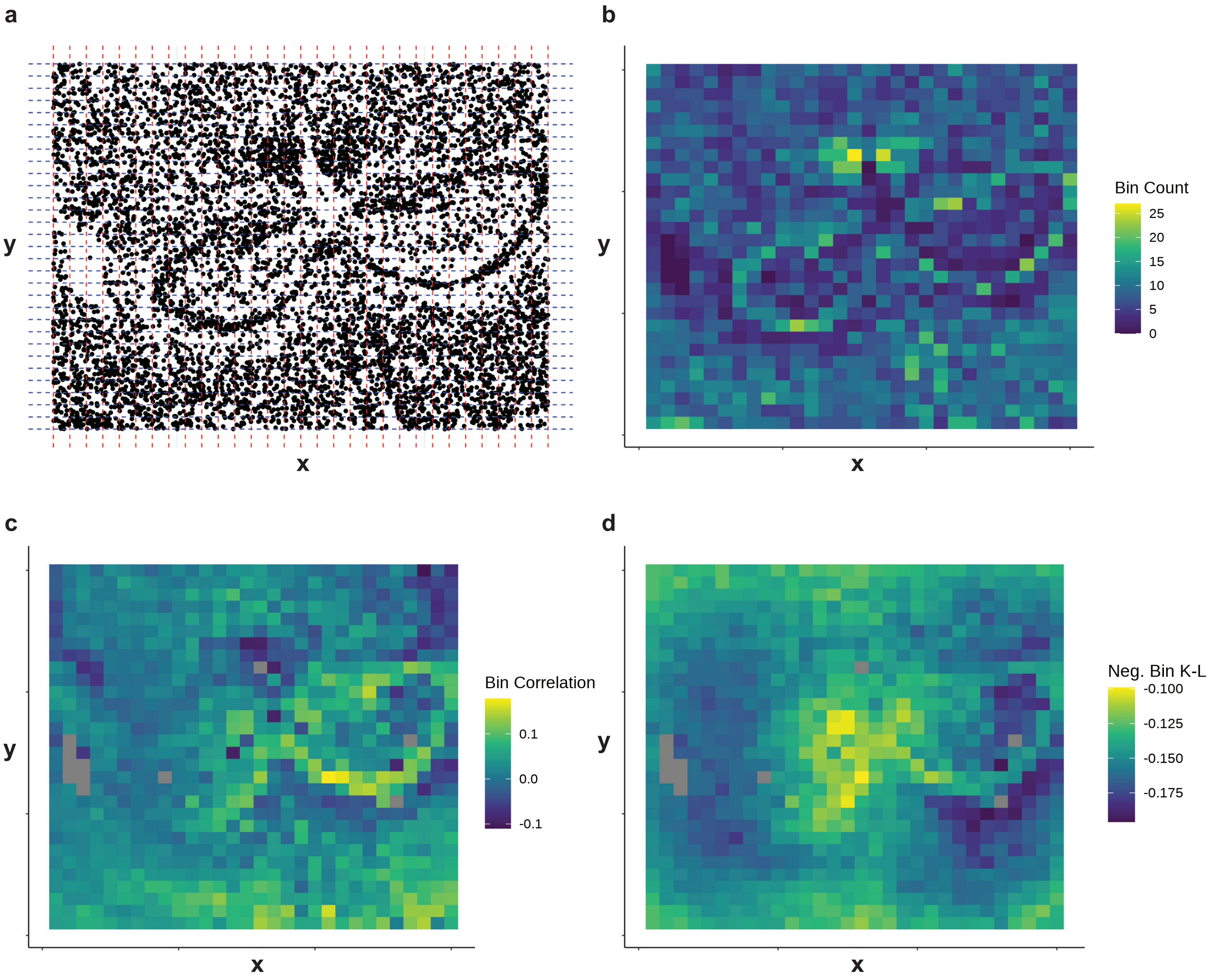
a
b
c
d
x
y
Bin Count
25
20
15
10
5
0
Bin Correlation
0.1
0.0
-0.1
Neg. Bin K-L
-0.100
-0.125
-0.150
-0.175

Figure S11. a, Binning of Vizgen data. The image region was binned with 30 by 30 bins on both axes. **b,** The number of cell centroid in each bin. Left open right closed intervals were applied. **c,** The mean Spearman correlation of between SMN graph distance and FR embedding distance between each cell pairs within each bin. **d,** The mean K-L Divergence of scHolography predictions for simulated mouse hippocampus data as ground truth within each bin.
