# Supplementary material for: scHolography: a computational method for single-cell spatial neighborhood reconstruction and analysis
Source: Genome Biol. 2024 Jun 24;25:164. doi: 10.1186/s13059-024-03299-3 (PMC11197379; doi:10.1186/s13059-024-03299-3)
Supplement: Supplementary file 3 — Additional file 3: Peer review history [file 13059_2024_3299_MOESM3_ESM.docx]

Review History

**First round of review**

**Reviewer 1**

**Were you able to assess all statistics in the manuscript, including the appropriateness of statistical tests used?**

**Yes:** The authors have provided a github repo containing all the analysis details.

**Were you able to directly test the methods?**

Yes

**Comments to author:**

My major concerns are the following:

1. Important details in the scHolography workflow are missing. The authors omitted the details of how ST and SC data are integrated and the details of Gale--Shapley for matching and Fruchterman--Reingold for graph construction. While FR is mostly for visualization, GS is critical for all the downstream results. I suggest that the authors add more detailed description of these steps and benchmark the performance of GS against other matching algorithms. The latter is particularly important as GS was originally designed for completely different settings that has little to do with geometry.

2. There is no clear evidence favoring 3D embedding. After reading the manuscript, I was left wondering what if one only uses Fruchterman--Reingold for 2D embedding and how the result 2D embedding would compare with other cell charting methods. I was not able to find convincing evidence in the manuscript that 3D embedding is necessary. Moreover, the distance comparison is in Figure 2e, 2i, etc. is not very meaningful, for the same reason as why one should not use distance in UMAP embedding space to quantify (dis)similarity.

3. There is no clear evidence that the findings enabled by scHolography cannot be achieved by deconvolution of spot-level spatial transcriptomics data. Given the approach requires ST data to start with, the use case of scHolography is not entirely clear. It would be much more convincing if the authors can demonstrate one case where scHolography can discover something that appropriate spot deconvolution cannot achieve.

**Reviewer 2**

**Were you able to assess all statistics in the manuscript, including the appropriateness of statistical tests used?**

No

**Were you able to directly test the methods?**

Yes

**Comments to author:**

This manuscript introduces scHolography, a novel framework for characterizing tissue spatial neighborhoods at single-cell resolution using scRNA-seq data. scHolography incorporates neural networks and the Gale-Shapley algorithm to learn 3D information from 2D spatial transcriptomics (ST) reference data and identify stable-matching neighbors (SMNs) for each cell. The major innovations of scHolography include the use of 3D coordinates to define spatial identity instead of 2D coordinates and the reconstruction of 3D tissue by projecting the cell-cell spatial connections onto an undirected SMN graph. The authors demonstrated the superior performance of scHolography in reconstructing spatial neighborhoods on both sequencing- and imaging-based ST datasets, including the 10X Xenium mouse hippocampus, 10X Visium mouse brain, Slide-seqV2 mouse hippocampus, and MERFISH anterior preoptic mouse brain.
Overall, scHolography looks to be a very promising method for spatial neighborhood reconstruction, with a significant improvement in accuracy over competing methods. However, the authors should address several critical issues regarding the design of the algorithm and the evaluation of the performance. First, the authors only use Seurat CCA as integration methods in performance evaluation. It is important to demonstrate the stability of scHolography by using alternative integration methods. Second, the authors compared scHolography's 3D reconstruction performance in one unmatching sample. Examining the broader applicability of scHolography in other situation would provide valuable insights. Third, the authors should interpret the result from a biological perspective. Additionally, scHolography assigns a fixed number of neighboring cells to each cell, which is inconsistent with the fact that different regions within a tissue can exhibit distinct cell densities. I have the following suggestions to improve the manuscript.
1. Integration: The initial step of scHolography involves integrating scRNA-seq data with ST data. However, it seems that the authors relied exclusively on Seurat CCA for this integration process. To ensure the robustness and reliability of the results, it is strongly recommended that the authors perform additional validation using alternative integration methods.
2. Benchmark: The authors discussed a limitation of 2D spatial cell charting methods in cases where there are mismatches between ST and SC data, as shown in Figure 2d. However, it is crucial for the authors to provide a more comprehensive evaluation of scHolography by comparing it with other existing tools using matched datasets. While Figure 2d highlights a specific instance of mismatch, a single comparison may not be sufficient to rigorously evaluate the performance of scHolography.
3. Robustness: In Figure 3d-f, the authors demonstrate scHolography's ability to reconstruct 3D tissue from a 2D reference, specifically using slice 400 as the reference. However, to further assess the robustness of scHolography, it is recommended that the authors present additional results using other slices as references. Including these supplementary results in the manuscript or as supplementary material would provide a more comprehensive understanding of scHolography's performance across different slices and validate its consistency in reconstructing 3D tissue structures from various reference points.
4. Interpretability of the algorithm. Are the most stable matching neighbors identified by scHolography interpretable? How can we interpret the result from a biological perspective?
5. Tissue heterogeneity: It is crucial for the authors to address the issue of tissue heterogeneity in their manuscript. Different regions within a tissue can indeed exhibit distinct cell densities, and this variation should be acknowledged. Currently, scHolography assigns a fixed number of neighboring cells (default 30) to each cell, which may not accurately capture the true spatial relationships in tissues with varying densities.

**Authors Response**

**Point-by-point responses to the reviewers’ comments:**

Reviewer #1: My major concerns are the following:
1. Important details in the scHolography workflow are missing. The authors omitted the details of how ST and SC data are integrated and the details of Gale--Shapley for matching and Fruchterman--Reingold for graph construction. While FR is mostly for visualization, GS is critical for all the downstream results. I suggest that the authors add more detailed description of these steps and benchmark the performance of GS against other matching algorithms. The latter is particularly important as GS was originally designed for completely different settings that has little to do with geometry.

We thank the reviewers for the insightful comments. We have added detailed description in Methods section for the integration of ST and SC data. We compared additional scHolography results when using LIGER (Welch et al., 2019), Harmony (Korsunsky et al., 2019), and fastMNN (Haghverdi et al., 2018). As shown in Figure 1, Seurat CCA integration resulted in the lowest mean K-L divergence and the highest mean correlation with the ground truth cell-cell distance for the simulated mouse hippocampus data. Using different integration methods, we also inspected the separation of CA1/CA2/CA3 hippocampus regions of the scHolography construction. Besides Seurat CCA, Harmony is the only method that successfully separates the three regions (Fig. 1g-l). Considering these results, we choose to use Seurat CCA integration as the default setting for our method default. We note, however, users can easily apply their preferred integration method before scHolography NN training and SMN graph construction. This gives the user flexibility if their datasets are better served by other integration methods.

 **Figure 1. a-d,** UMAP plot of Seurat CCA, Harmony, LIGER, and fastMNN integration of scRNA-seq and ST data. **e,** KL-divergence of scHolography predictions powered by different integration methods for simulated mouse hippocampus data as ground truth. **f,** Spearman correlation between scHolography cell-cell SMN distance powered by different integration methods and simulated mouse hippocampus cell-cell distance. **g-i,** Visualization of Harmony, LIGER, and fastMNN powered scHolography prediction results. **j-l,** Comparison of Harmony, LIGER, and fastMNN powered scHolography reconstruction results for CA1, CA2, and CA3 cell distance to CA3 cells. Cell distances were normalized for each method by the mean distance between CA3 cells and CA3 cells.

We also appreciate the insightful comments about the use of GS in our workflow. We note that it is our innovation to use GS algorithm to reconstruct single-cell spatial neighborhoods. The GS method pairs up subjects based on their preferences and ensures the stability of matching. Under the assumption that the SC sample comes from the same piece of tissue and the data contains the true spatial neighbors of cells, we apply this stable matching idea to single cells to assign each cell with other cells as spatial neighbors based on their predicted spatial affinity as the preference such that no cells will prefer other cells over their assigned neighbors. This application will ensure the optimal assignment of single cells and no two cells will occupy the same spot. To the best of our knowledge, among matching algorithms, the GS algorithm is the widely used one that achieves this stable matching task. Other matching methods, such as maximum weight matching like the Hungarian algorithm, can also solve assignment problems (Fredman & Tarjan, 1987). However, the GS is more efficient with a polynomial time complexity O(n²) than the complexity O(n^3^) of the Hungarian algorithm, where n is the number of total cells (Pavlopoulos et al., 2018). Furthermore, we have extensively tested scHolography with the application of GS algorithm and demonstrated the efficacy of the performance. We acknowledge that future development in cell assignment could further improve our algorithm, and we have added this point into Discussion.

2. There is no clear evidence favoring 3D embedding. After reading the manuscript, I was left wondering what if one only uses Fruchterman--Reingold for 2D embedding and how the result 2D embedding would compare with other cell charting methods. I was not able to find convincing evidence in the manuscript that 3D embedding is necessary. Moreover, the distance comparison is in Figure 2e, 2i, etc. is not very meaningful, for the same reason as why one should not use distance in UMAP embedding space to quantify (dis)similarity.

We thank the reviewer for raising this important question with regard to 3D embedding. One of the main goals for us to develop scHolography for 3D tissue reconstruction and analysis is to study cell-cell interaction and microenvironment that exist in 3D rather than 2D. In contrast, most existing cell charting tools, such as CellTrek and CytoSPACE, assign single cells and their associated transcriptome back to 2D space that is defined by spatial transcriptomic results. It is also important to note that 2D projection or embedding assumes that all single cells are derived from the same 2D slice, which is not the case. Thus, we believe that it is an innovative and important step forward to analyze single-cell and spatial transcriptomic data in 3D. To demonstrate the feasibility and utility of our approach, we show: 1) scHolography can learn 3D information from 2D spatial transcriptomic reference; 2) 3D assignment performs better than 2D assignment in benchmarks; 3) 3D based results provide biologically meaningful information. First, in the revised manuscript, we have further confirmed that scHolography effectively learns 3D information from 2D tissue slices by learning from a single slice of multiple serial sections and reconstructing the tissue (new Fig. S5-6 in the manuscript). In revision, we have additionally applied scHolography to a mouse kidney dataset, where 3D tissue organization is well appreciated. By learning from a 2D spatial reference slice, our scHolography reconstruction has corrected identified xx cell populations that are organized in 3D rather than 2D (new Fig. 7 in the manuscript). This real-world result further highlights the ability to learn 3D information from a 2D tissue slice. Second, we show that scHolography measures the SMN distance between different cell types, quantified their layered organization and evaluated the global spatial reconstruction quality by using K-L divergence – a rigorous approach allowing statistical tests, rather than eyeballing the similar or different cell organizations in our reconstruction. Thus, our method is built on quantitative evaluations of tissue organization. Third, our neighborhood analysis integrates the single-cell transcriptome of all cells from a given neighborhood into a spatially defined “mini-bulk” transcriptome. This approach has significantly enhanced our ability to 1) demarcate spatial cell clusters by not only the cell type composition but also their collective transcriptome; 2) identify key marker genes, many of which are important signaling molecules, by using the mini-bulk transcriptome for functional analysis; 3) examine cell-cell interactions with spatially defined ligand-receptor analysis. Finally, in response to the reviewer’s comment, we have embedded our results in 2D rather than 3D as shown in Figure 2b below. Such embedding loses layered tissue organization of the foreskin sample and performs poorly as judged by Spearman Correlation (Fig. 2c). Together, we hope the reviewer would agree that scHolography is a 3D visualization method. It translates high-dimensional spatial transcriptomics data into actionable insights about tissue organization and cellular microenvironment.

**Figure 2. a,** Spearman correlation between scHolography inferred cell-cell SMN distance and the ground-truth cell-cell spatial distance with cell-cell embedding distance on 2D and 3D PCA and UMAP embeddings. **b,** 2D FR embeddings of human skin. **c,** Spearman correlation between SMN graph distance and 2D and 3D FR embedding distance between cell pairs.

For the spatial analysis based on the SMN distance, we note that the core of our method is to construct a stable matching neighbor (SMN) graph of cells. To get the SMN distance, we first used the real measurement of pixel-pixel distance from ST datasets to generate the SICs. We next used a machine learning approach to learn the transcriptome-to-distance transformation. Finally, we used the GS algorithm to identify the SMN of each query cell. Because of these transformations, we prefer not to refer the SMN distance as the spatial distance. However, unlike dimensionality reduction methods widely used for transcriptome analysis, the SMN distance is a derivative from the spatial distance, and it describes the spatial relationship among cells such that the shorter SMN distance indicates the closer relationship between cells. Our existing data of spatial neighborhood analysis from human foreskin samples recapitulated experimental observations of the layered organization of distinct epithelial and dermal cells by using the SMN distance. To further demonstrate that the SMN distance is meaningful in capturing biological tissue structure, we made a new comparison of the correlation between scHolography inferred cell-cell SMN distance and the ground-truth cell-cell spatial distance with cell-cell embedding distance on 2D and 3D PCA and UMAP embeddings (Fig. 2a). The SMN distance used by scHolography significantly outperforms the other cell-cell embedding distance, validating our approach.

3. There is no clear evidence that the findings enabled by scHolography cannot be achieved by deconvolution of spot-level spatial transcriptomics data. Given the approach requires ST data to start with, the use case of scHolography is not entirely clear. It would be much more convincing if the authors can demonstrate one case where scHolography can discover something that appropriate spot deconvolution cannot achieve.

To compare the performance between scHolography and spot deconvolution-based method, we have applied SPOTlight decomposition (Elosua-Bayes M et al., Nucleic Acids Research 2021, cited by 324) to human dermal cells, which we have already analyzed with scHolography. As shown in Figure 3a-b below, SPOTlight decomposition resolves each spot into several cell types based on the mixed transcriptome. Next, because SPOTlight decomposition doesn’t have a spatial neighborhood analysis function, we used the “BuildNicheAssay” approach, described by Seurat V5, to identify 4 dermal niches (Fig. 3c). Finally, we identified enriched gene expression in each spatial niche (Fig. 3d). In contrast to our result from scHolography, SPOTlight decomposition-based approach didn’t identify distinct dermal cell neighborhood, judging by the enriched genes from each neighborhood. This result was in contrast to our identification of 4 experimentally validated, distinct neighborhoods (see Fig. 6c-e in the manuscript). For example, as we showed in our human skin dermal spatial neighborhood analysis, our method can identify spatial neighborhoods of papillary and reticular dermal cells as well as RGS5+ pericytes, which were experimentally validated (Philippeos et al., 2018). When we did the niche analysis with SPOTlight decomposition for the same number of neighborhoods, no such observation can be made.

To understand why scHolography outperforms SPOTlight decomposition or spot deconvolution methods in general, we examined more deeply how each method reconstructs the spatial neighbor based on single cell and spatial data. We note that some fundamental differences exist between our approach and decomposition methods. First, decomposition methods break down the composition of each spatial pixel by single-cell types but not single cells. Thus, for dermal cells in different spatial pixels, the identical, cell type-based transcriptome is used, and the spatial niche is defined by the differences in the composition of discrete cell types. This approach loses the resolution in the single-cell transcriptome. Second, the overall strategy of decomposition methods is to treat spatial data as the query and single-cell data as the reference. As outlined above, this approach loses transcriptome heterogeneity detected by high-dimensional single-cell data. In contrast, scHolography uses single-cell data as the query and spatial data as the reference. We can make inferences on single cells with a higher resolution. Distinct from other cell charting methods, scHolography further captures spatial patterns by using a high-dimensional distance matrix of pairwise pixel-pixel distances from spatial registration. After neural network training and stable matching neighbor assignment, scHolography reconstructs the tissue organization with a much higher resolution and precision, as demonstrated by the spatial gene gradient as well as the identification of distinct spatial neighborhoods that are experimentally detected by previous studies. We have added this line of discussion in the revised manuscript, and we thank the reviewer for this great suggestion.

**Figure 3. a,** SPOTlight deconvolution results for human skin 10X Visium data. **b,** Defined dermal cell-containing spatial spots. **c,** Spatial niche analysis for dermal cell-containing spatial spots. **d,** Heatmap of top differentially expressed genes for each spatial niche.

Reviewer #2: This manuscript introduces scHolography, a novel framework for characterizing tissue spatial neighborhoods at single-cell resolution using scRNA-seq data. scHolography incorporates neural networks and the Gale-Shapley algorithm to learn 3D information from 2D spatial transcriptomics (ST) reference data and identify stable-matching neighbors (SMNs) for each cell. The major innovations of scHolography include the use of 3D coordinates to define spatial identity instead of 2D coordinates and the reconstruction of 3D tissue by projecting the cell-cell spatial connections onto an undirected SMN graph. The authors demonstrated the superior performance of scHolography in reconstructing spatial neighborhoods on both sequencing- and imaging-based ST datasets, including the 10X Xenium mouse hippocampus, 10X Visium mouse brain, Slide-seqV2 mouse hippocampus, and MERFISH anterior preoptic mouse brain.
Overall, scHolography looks to be a very promising method for spatial neighborhood reconstruction, with a significant improvement in accuracy over competing methods. However, the authors should address several critical issues regarding the design of the algorithm and the evaluation of the performance. First, the authors only use Seurat CCA as integration methods in performance evaluation. It is important to demonstrate the stability of scHolography by using alternative integration methods. Second, the authors compared scHolography's 3D reconstruction performance in one unmatching sample. Examining the broader applicability of scHolography in other situation would provide valuable insights. Third, the authors should interpret the result from a biological perspective. Additionally, scHolography assigns a fixed number of neighboring cells to each cell, which is inconsistent with the fact that different regions within a tissue can exhibit distinct cell densities. I have the following suggestions to improve the manuscript.

We thank the reviewer’s insightful comments and suggestions that help us to strengthen our manuscript. In revision, we have addressed all these issues in full and significantly improved our manuscript.
1.      Integration: The initial step of scHolography involves integrating scRNA-seq data with ST data. However, it seems that the authors relied exclusively on Seurat CCA for this integration process. To ensure the robustness and reliability of the results, it is strongly recommended that the authors perform additional validation using alternative integration methods.

We thank the reviewer for the suggestion of testing different integration methods for scHolography. To do so, we compared additional scHolography results when using LIGER (Welch et al., 2019), Harmony (Korsunsky et al., 2019), and fastMNN (Haghverdi et al., 2018). As shown in Figure 1 below, Seurat CCA integration resulted in the lowest mean K-L divergence (Fig. 1e) and the highest mean correlation with the ground truth cell-cell distance for the simulated mouse hippocampus data (Fig. 1f). Using different integration methods, we also inspected the separation of CA1/CA2/CA3 hippocampus regions of the scHolography construction. Besides Seurat CCA, Harmony is the only method that successfully separates the three regions (Fig. 1g-l). Considering these results, we choose to use Seurat CCA integration as the default setting for our method default. We note, however, users can easily apply their preferred integration method before scHolography NN training and SMN graph construction. This gives the user flexibility if their datasets are better served by other integration methods.

**Figure 1. a-d,** UMAP plot of Seurat CCA, Harmony, LIGER, and fastMNN integration of scRNA-seq and ST data. **e,** KL-divergence of scHolography predictions powered by different integration methods for simulated mouse hippocampus data as ground truth. **f,** Spearman correlation between scHolography cell-cell SMN distance powered by different integration methods and simulated mouse hippocampus cell-cell distance. **g-i,** Visualization of Harmony, LIGER, and fastMNN powered scHolography prediction results. **j-l,** Comparison of Harmony, LIGER, and fastMNN powered scHolography reconstruction results for CA1, CA2, and CA3 cell distance to CA3 cells. Cell distances were normalized for each method by the mean distance between CA3 cells and CA3 cells.

2.      Benchmark: The authors discussed a limitation of 2D spatial cell charting methods in cases where there are mismatches between ST and SC data, as shown in Figure 2d. However, it is crucial for the authors to provide a more comprehensive evaluation of scHolography by comparing it with other existing tools using matched datasets. While Figure 2d highlights a specific instance of mismatch, a single comparison may not be sufficient to rigorously evaluate the performance of scHolography.

To evaluate scHolography with more cases, we first added a new analysis to conduct a simulated mouse hippocampus study with a region-matched 10X Visium dataset. We subset the 10X Visium data to include only the region surrounding the hippocampus. Similar to our conclusion from the unmatched region simulation study in our original manuscript, scHolography has the best overall performance as determined by low K-L Divergence, high cosine similarity, and robust separation of the CA1/CA2/CA3 regions, in comparison to CellTrek, CytoSPACE, Seurat and Tangram (Fig.2). We have added these new results as new Figure S3 to the revised manuscript.

**Figure 2. a,** Illustration of 10X Visium mouse hippocampus ST reference region. **b,** Illustration of mouse hippocampus simulated SC query region. **c,** KL-divergence of spatial cell charting method predictions for simulated mouse hippocampus data as ground truth. **d,** Heatmap for the mean of cosine similarity between method-predicted spatial neighborhood accumulated expression and simulated mouse hippocampus spatial neighborhood accumulated expression. The size of the neighborhood varies from 3 to 15 cells. **e,** Visualization of scHolography, Celltrek, CytoSPACE, Seurat, and Tangram single-cell spatial charting results of a mouse hippocampus data. **f,** Comparison of scHolography, Celltrek, CytoSPACE, Seurat, and Tangram results for predicted CA1, CA2, and CA3 cell distance to CA3 cells. Cell distances were normalized for each method by the mean distance between CA3 cells and CA3 cells.

To further benchmark the performance of scHolography in tissues different than brain and skin, we applied scHolography to a mouse kidney dataset (Park et al., 2018) and compared its performance against current methods (Fig. 3). Notably, the ability of 3D tissue reconstruction of scHolography recapitulated kidney spatial organization (Fig. 3c-d), which have been revealed by morphological analysis (Ransick et al., 2019). These results are in contrast to the result from CellTrek (Fig. 3e), CytoSPACE (Fig. 3f), Seurat (Fig. 3g) and Tangram (Fig. 3h). Moreover, our scHolography spatial neighborhood analysis successfully identified the spatial heterogeneity among Proximal Tubule (PT) cells of mouse kidney, where PT_1 is closer to Distal Convoluted Tubule (DCT) cells and PT_2 is closer to Loop of Henle (LOH) cells (Fig. 3i-k). Finally, scHolography also identified spatially enriched genes from PT_1 and PT_2 neighborhoods, which should allow validation and functional studies (Fig. 3j). Altogether, these additional benchmarks and analyses have further demonstrated the utility of scHolography and the improvement over current leading methods. We have added the new matched study to the manuscript as Fig. S3 and the kidney study to the manuscript as new Fig. 7.

**Figure 3. a,** H&E image of mouse kidney coronal section. **b,** UMAP plot of mouse kidney scRNA-seq data. **c,** 3D visualization of mouse kidney scHolography reconstruction result. **d,** Boxplots for scHolography predicted PT, DCT, CD-IC, CD-PC and LOH cell distance to CD-PC and LOH cells. Cell distances were normalized for each method by the mean distance from CD-PC and LOH cells to the two cell types. **e-h,** Comparison of Celltrek, CytoSPACE, Seurat, and Tangram mouse kidney prediction results. Left panel: Visualization of prediction outcome; Right panel: Predicted PT, DCT, CD-IC, CD-PC and LOH cell distance to CD-PC and LOH cells. Cell distances were normalized for each method by the mean distance from CD-PC and LOH cells to the two cell types. **i,** Spatial neighborhood analysis for mouse kidney PT cells. Two distinct neighborhoods are identified based on the clustering of the accumulated expression profile of PT SMNs. **j,** Heatmap of top marker genes for PT_1 and PT_2 accumulated SMN expression profile. k, Violin plot of SMN distance between two PT spatial neighborhood cells and DCT cells (left). Violin plot of SMN distance between two PT spatial neighborhood cells and LOH cells (right).

3.      Robustness: In Figure 3d-f, the authors demonstrate scHolography's ability to reconstruct 3D tissue from a 2D reference, specifically using slice 400 as the reference. However, to further assess the robustness of scHolography, it is recommended that the authors present additional results using other slices as references. Including these supplementary results in the manuscript or as supplementary material would provide a more comprehensive understanding of scHolography's performance across different slices and validate its consistency in reconstructing 3D tissue structures from various reference points.

We thank the reviewer for this suggestion to demonstrate the robustness of scHolography. We used additional Merfish data (Moffitt et al., 2018) and different slices as references for scHolography analysis. Specifically, we took Samples 1 and 2 from Animal 1 at the central z-plane. The centroids of cells were calculated using the terra package. 1500 cells were subsampled from each slice of Samples 1 and 2. To reconstruct a stacked 2D structure of Samples 1 and 2, PASTE package (Zeira et al., 2022) was used to conduct pairwise slice alignment for slices of each sample. Data was normalized by Seurat SCTransform. Cells from 6 slices of Sample 1 were reconstructed twice using slice 10 or slice 500 as the reference, respectively. Similarly, cells from 5 slices of Sample 2 were reconstructed twice using slice 620 or slice 1020 as the reference, respectively.

**Figure 4. a,** Stacked-2D plot of Merfish mouse cortex sample 1 (slice 10, 100, 210, 310, 400, and 500) colored by the slice. b, Stacked-2D plot of Merfish mouse cortex sample 1 colored by cell types. **c-d,** 3D visualization of Merfish sample1 scHolography reconstruction result. **c,** use slice 10 as the reference; **d,** use slice 500 as the reference. **e-f,** SMN distances from L2/3 IT, L4/5 IT, L5 IT, L6 IT to L6 IT in Merfish sample1 scHolography prediction. **e,** use slice 10 as the reference; **f,** use slice 500 as the reference. **g-h,** SMN distances from slice 10, 100, 210, 310, 400, and 500 to slice 10 in Merfish sample 1 scHolography prediction. **g,** use slice 10 as the reference; **h**, use slice 500 as the reference.

**Figure 5. a,** Stacked-2D plot of Merfish mouse cortex sample 2 (620, 710, 810, 910, and 1020) colored by the slice. **b,** Stacked-2D plot of Merfish mouse cortex sample colored by cell types. **c-d,** 3D visualization of Merfish sample2 scHolography reconstruction result. **c,** use slice 620 as the reference; **d,** use slice 1020 as the reference. **e-f,** SMN distances from L2/3 IT, L4/5 IT, L5 IT, L6 IT to L6 IT in Merfish sample2 scHolography prediction. **e,** use slice 620 as the reference; **f,** use slice 1020 as the reference. **g-h,** SMN distances from slice 620, 710, 810, 910, and 1020 to slice 620 in Merfish sample 2 scHolography prediction. **g,** use slice 620 as the reference; **h,** use slice 1020 as the reference.

As shown Figures 4-5, we demonstrated the consistent performance of scHolography by using either the bottom or top slice as the reference for both samples. These additional studies have further validated scHolography’s ability to learn 3D information from 2D slices and reconstruct tissue organization. We have added these results as new Fig. S5 and S6 in revision.

4.      Interpretability of the algorithm. Are the most stable matching neighbors identified by scHolography interpretable? How can we interpret the result from a biological perspective?

The main goal for developing scHolography is to learn information about tissue microenvironment, perform quantitative analysis of cell neighborhood, and identify spatially enriched genes and gene gradients across tissues. To do so, we need a quantitative measurement of cell-cell distance. In our method, we construct a stable matching neighbor (SMN) graph of cells as the measurement. To get the SMN distance, we first used the real measurement of pixel-pixel distance from ST datasets to generate the SICs. We next used a machine learning approach to learn the transcriptome-to-distance transformation. Finally, we used the GS algorithm to identify the SMN of each query cell. Because of these transformations, we prefer not to refer the SMN distance as the spatial distance. However, the SMN distance is a derivative from the spatial distance, and it describes the spatial relationship among cells such that the shorter SMN distance indicates the closer relationship between cells. By using the information gained from the stable matching neighbors, we have shown that tissue composition analysis by cell type (such as Fig. 4h, Fig. 6b and Fig. 8e) provides quantitative information about tissue microenvironment. These data also allow quantitative evaluation of similarities and differences between different cell neighborhoods. Because our reconstruction generates results of both cell composition and single-cell transcriptome, this allows the identification of spatially enriched genes in each neighborhood (such as Fig. 5d, Fig. 6c, Fig. 7j, and Fig. 8f). We note these spatially enriched genes are identified through numerous single cells, permitting statistical evaluation. This is particularly valuable for experimental biologists who often make observations based on specific antibody staining or in situ hybridization for a few selected markers but are unable to quantify such observations and evaluate the significance in a microenvironment. Finally, scHolography also allows gene gradient identification across tissues (such as Fig. 5d and Fig. 6f). We have added some of these discussions about the SMN in revision.

5.      Tissue heterogeneity: It is crucial for the authors to address the issue of tissue heterogeneity in their manuscript. Different regions within a tissue can indeed exhibit distinct cell densities, and this variation should be acknowledged. Currently, scHolography assigns a fixed number of neighboring cells (default 30) to each cell, which may not accurately capture the true spatial relationships in tissues with varying densities.

The reviewer raised an important question about tissue heterogeneity and its potential impact on the performance of scHolography. To provide more information, we determined the performance of scHolography when using different numbers of neighbors (Fig. 6).

**Figure 6** Spearman correlation of cell-cell scHolography SMN distance to the simulated mouse hippocampus data, changing the parameter # of neighbor for scHolography reconstruction from 2 to 100. Red line: default= 30.

For dense tissue like skin, the increasing number of neighboring cells generally improves the performance of scHolography. In addition, in our new study on the simulated mouse hippocampus data, we found that scHolography performed better in high-density regions than low-density regions (Fig. 7). Although the current version of scHolography does not have the ability to adjust the number of neighbors within one experiment, users can test different numbers of neighbors to accommodate heterogeneous tissue density. The default number of 30 works well for the tissues we have tested, including relative low-density tissues, such as brain, and relative high-density tissues, such as skin, or kidney. We also note that the variation in tissue density is an important issue in the field of spatial transcriptomics, which is affected or controlled by many biological properties of the cell, including but not limited to cell size, adhesion, migration and tissue heterogeneity. Future work will be needed to fully address these issues. We provide a discussion about the issue in the revised manuscript.

**Figure 7. a**, Binning of Vizgen data. The image region was binned with 30 by 30 bins on both axes. **b,** The number of cell centroid in each bin. Left open right closed intervals were applied. **c,** The mean Spearman correlation of between SMN graph distance and FR embedding distance between each cell pairs within each bin. **d,** The mean K-L Divergence of scHolography predictions for simulated mouse hippocampus data as ground truth within each bin.

**Reference**

Fredman, M. L., & Tarjan, R. E. (1987). Fibonacci heaps and their uses in improved network optimization algorithms. *Journal of the ACM (JACM)*, *34*(3), 596-615.

Haghverdi, L., Lun, A. T., Morgan, M. D., & Marioni, J. C. (2018). Batch effects in single-cell RNA-sequencing data are corrected by matching mutual nearest neighbors. *Nature biotechnology*, *36*(5), 421-427.

Korsunsky, I., Millard, N., Fan, J., Slowikowski, K., Zhang, F., Wei, K., Baglaenko, Y., Brenner, M., Loh, P. R., & Raychaudhuri, S. (2019). Fast, sensitive and accurate integration of single-cell data with Harmony. *Nat Methods*, *16*(12), 1289-1296. <https://doi.org/10.1038/s41592-019-0619-0>

Moffitt, J. R., Bambah-Mukku, D., Eichhorn, S. W., Vaughn, E., Shekhar, K., Perez, J. D., Rubinstein, N. D., Hao, J., Regev, A., Dulac, C., & Zhuang, X. (2018). Molecular, spatial, and functional single-cell profiling of the hypothalamic preoptic region. *Science*, *362*(6416). <https://doi.org/10.1126/science.aau5324>

Park, J., Shrestha, R., Qiu, C., Kondo, A., Huang, S., Werth, M., Li, M., Barasch, J., & Suszták, K. (2018). Single-cell transcriptomics of the mouse kidney reveals potential cellular targets of kidney disease. *Science*, *360*(6390), 758-763. <https://doi.org/10.1126/science.aar2131>

Pavlopoulos, G. A., Kontou, P. I., Pavlopoulou, A., Bouyioukos, C., Markou, E., & Bagos, P. G. (2018). Bipartite graphs in systems biology and medicine: a survey of methods and applications. *Gigascience*, *7*(4), 1-31. <https://doi.org/10.1093/gigascience/giy014>

Philippeos, C., Telerman, S. B., Oulès, B., Pisco, A. O., Shaw, T. J., Elgueta, R., Lombardi, G., Driskell, R. R., Soldin, M., Lynch, M. D., & Watt, F. M. (2018). Spatial and Single-Cell Transcriptional Profiling Identifies Functionally Distinct Human Dermal Fibroblast Subpopulations. *J Invest Dermatol*, *138*(4), 811-825. <https://doi.org/10.1016/j.jid.2018.01.016>

Ransick, A., Lindström, N. O., Liu, J., Zhu, Q., Guo, J. J., Alvarado, G. F., Kim, A. D., Black, H. G., Kim, J., & McMahon, A. P. (2019). Single-Cell Profiling Reveals Sex, Lineage, and Regional Diversity in the Mouse Kidney. *Dev Cell*, *51*(3), 399-413.e397. <https://doi.org/10.1016/j.devcel.2019.10.005>

Welch, J. D., Kozareva, V., Ferreira, A., Vanderburg, C., Martin, C., & Macosko, E. Z. (2019). Single-Cell Multi-omic Integration Compares and Contrasts Features of Brain Cell Identity. *Cell*, *177*(7), 1873-1887.e1817. <https://doi.org/10.1016/j.cell.2019.05.006>

Zeira, R., Land, M., Strzalkowski, A., & Raphael, B. J. (2022). Alignment and integration of spatial transcriptomics data. *Nat Methods*, *19*(5), 567-575. <https://doi.org/10.1038/s41592-022-01459-6>

**Second round of review**

**Reviewer 1**

The authors have addressed most of my concerns from the previous round review. However, it is still not clear to me why 3D tissue structures can be learned from 2D sections by the proposed method. From an information theory perspective, the only possibility is that the 2D section used in training the NN weights already contains the "3D" structure in the point cloud the method produces. In this sense, these are not 3D structures of the tissue, but rather 3D representation of the spatial neighboring information of different cell populations in the 2D section. Therefore, I strongly suggest that the authors do not make the overly strong claim that "scHolography learns 3D information from 2D references" since the 3D (point cloud) and 2D (tissue) spaces are not comparable.

**Reviewer 2**

I really appreciate the authors' exceptional efforts in thoroughly addressing all of my detailed remarks. I support the publication of this paper.

**Authors Response**

**Point-by-point responses to the reviewers’ comments:**

Specifically to the remaining point raised by reviewer #1, we have revised the text to state that our method generates 3D spatial representation or infer 3D tissue organization, instead of the strong statement “scHolography learns 3D information from 2D references”, throughout the manuscript.
